# Molecular detection and identification of relapsing fever *Borrelia* in ticks and wild small mammals in China

**DOI:** 10.1080/22221751.2022.2134054

**Published:** 2022-11-04

**Authors:** Xiao-Ai Zhang, Feng Tian, Yue Li, Xiao-Long Zhang, Bao-Gui Jiang, Bao-Cheng Liu, Jing-Tao Zhang, Shen Tian, Heng Ding, Shuang Li, Hao Li, Li-Qun Fang, Wei Liu

**Affiliations:** a State Key Laboratory of Pathogen and Biosecurity, Beijing Institute of Microbiology and Epidemiology, Beijing, People’s Republic of China; b Urumqi Customs Port Outpatient Department, Xinjiang International Travel Health Care Center, Urumqi, People’s Republic of China; c Science and Technology Research Center of China Customs (STRC), Beijing, People’s Republic of China; d School of Public Health, Guangzhou Medical University, Guangzhou, People’s Republic of China; e School of Public Health, Peking University, Beijing, People’s Republic of China

**Keywords:** Relapsing fever, *Borrelia*, tick, mammal, China

## Abstract

We identified relapsing fever (RF) *Borrelia* in 1.45% (145/10426) of the ticks and 1.40% (40/2850) of the wild mammals in a field investigation in China. Three RF *Borrelia* species, including human-pathogenic *Borrelia miyamotoi*, *Borrelia persic*a and unclassified *Babesia* sp. were determined. Main species determined from ticks was *B. miyamotoi* (44.14%), followed by the unclassified *Borrelia* sp. (42.76%), and *Borrelia theileri* (13.10%). In wild mammals, main species found was *B. persica* (57.50%), followed by the unclassified *Borrelia* sp. (40.00%), and *B. miyamotoi* (2.50%). We determined *B. theileri* and *B. persica* in China for the first time*.* The coexistence of RF *Borrelia* species in one tick species in a given region was observed, with the most frequent coexistence seen for *B. miyamotoi* and the unclassified *Borrelia* sp. in *Dermacentor silvarum, Haemaphysalis japonica, Haemaphysalis longicornis, and Ixodes persulcatuss* respectively. The wide distribution and high variety of RF *Borrelia* in China pose a potential threat to public health.

**Dear Editor,**

Relapsing fever (RF) is a zoonosis caused by the relapsing fever group spirochetes of the genus *Borrelia* that have been distributed throughout Africa, Central Asia, North America and Southern Europe [1]. Typically, RF *Borrelia* is transmitted by soft ticks, with the exception of the human louse-borne *Borrelia recurrentis*, *Borrelia lonestari* and *Borrelia miyamotoi* harboured by hard ticks [2–4]. Initially distinguished by geography and vector types, the RF *Borrelia* used to be arbitrarily grouped into the Old World (Palearctic-Afrotropic ecozone) and the New World (Nearctic ecozone) *Borreliae* [2]. With recently increasing report of novel species, such as *Candidatus* Borrelia javanense*,* in total 27 RF *Borrelia* species spirochetes have been identified to date [5]. At least three RF *Borrelia* species have been documented in China, including the identification of *B. miyamoti* in Heilongjiang; *Candidatus* Borrelia javanense in ticks in Guangxi; *Candidatus* Borrelia fainii in bats in Hubei and Shandong according to the recent studies [5–8]. Generally, reports on human disease are few and the studies were limited to the northeastern region. The current study was designed to determine the molecular evidence of RF *Borrelia* in mammal hosts and ticks in a wide region in China, further exploring the molecular diversity in relation to the vector and host species.

From 2017 to 2021, host-seeking ticks were collected by flagging over vegetation, and wild small mammals were captured by snap traps from 11 provinces in six eco-climate regions in China [9] (Figure 1(A)). The species of tick and small mammals were identified by morphology and further confirmed by sequencing of mitochondrial 16S ribosomal DNA (16S rDNA) and cytochrome b gene respectively (Table S1). Genomic DNA was extracted from ticks and mammal tissues using AllPrep DNA/RNA mini kit (Qiagen, Germany), which was screened for RF *Borrelia* using a touchdown PCR targeting the 353-bp 16S rDNA gene (*rrs*) [10] (Table S2). The positive samples were further characterized by amplifying and sequencing *rrs*, flagellin (*flaB*), and glycerophospho diester phosphodiesterase (*glpQ*) genes [6,7,10–13] (Table S2). The prevalence (95% confidence intervals) was calculated by maximum likelihood estimation using the program PooledInfRate. Phylogenetic tree was established using the maximum-likelihood method with the GTRGAMMA model in RAxML. Bootstrap values were calculated with 1000 replicates. The sequences generated in this study were submitted to GenBank under the accession numbers ON059648∼ON059658, ON209364∼ON209371, ON365959∼ON365960, ON059603-ON059643, ON060662∼ON060671, ON113496∼ON113497, ON184022∼ON184029, ON361163∼ ON361170 ON148112∼ON148132 (Table S3). The study protocol was approved by the Ethics Committee of Beijing Institute of Microbiology and Epidemiology.

**Figure 1.**
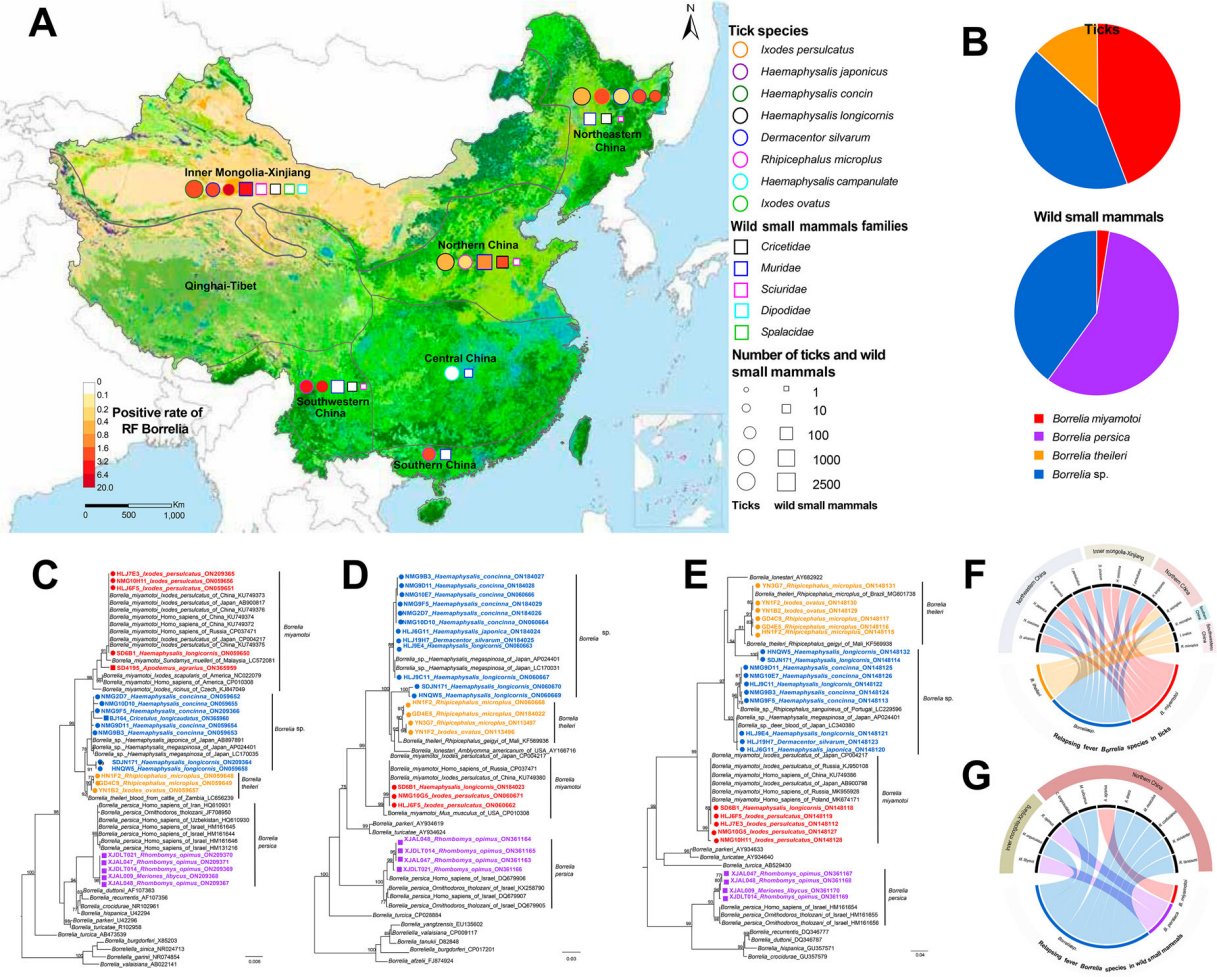
The distribution and genetic characterization of relapsing fever *Borrelia* in ticks and wild small mammals in China. (A) Geographic distribution of Relapsing Fever (RF) *Borrelia* species in China. The collection site for the sampling and test of RF *Borrelia*. Eco-climate regions were used for the geographic description, with six of them sampled: Northeastern China, Inner Mongolia-Xinjiang, Northern China, Central China, Southwest China and Southern China. The colour of the circles indicates positive rate of RF *Borrelia* in ticks; the colour of circle outlines represents tick species; the size of circle represents the number of ticks collected in the area. The colour of the squares indicates positive rate of RF *Borrelia* in wild small mammals; the colour of square outlines represents families of wild small mammals; the size of square represents the number of wild small mammals collected in the area. (B) The RF *Borrelia* species determined in ticks and wild small mammals. Phylogenetic analysis of RF *Borrelia* species based on *rrs* (C), *flaB* (D) and *glpQ* (E) genes. The tree was constructed by using the maximum-likelihood method with the GTRGAMMA model in RAxML. Bootstrap values were calculated with 1000 replicates. Scale bar indicates the degree of divergence represented by a given length of branch. RF *Borrelia* species determined in the current study were shown in colour. Sequences from ticks and wild small mammals were labelled with circle and square respectively. Chord diagrams between RF *Borrelia* species and tick species (F) and wild small mammals (G) in five eco-climate regions in China.

A total of 10,426 ticks that belonged to 4 genus and 8 species were collected, which were grouped into 4268 pools by species and locations for further tests (Figure 1(A)). In total, positive detection of RF *Borrelia* was determined in 1.45% (1.23–1.74%) of the ticks of 7 species, including 4.76% of *Ixodes ovatus*, 3.42% of *Ixodes persulcatus*, 2.81% of *Haemaphysalis japonica*, 2.13% of *Haemaphysalis concinna*, 1.74% of *Rhipicephalus microplus*, 0.85% of *Dermacentor silvarum*, and 0.63% of *Haemaphysalis longicornis* (Table S4). The positive detection was obtained from five of six sampled eco-climate regions, with the highest rate observed in Southwestern China (5.58%), followed by Inner Mongolia-Xinjiang (2.37%), Southern China (2.19%), Northeastern China (1.20%), and Northern China (0.59%) (Table S4).

Totally 2850 wild small mammals that belonged to 50 species in 5 families were tested using spleen samples (Figure 1A). Altogether, 40 out of 2850 (1.40%) mammals of 11 species were positive for RF *Borrelia*, including in 25.68% (19/74) of *Rhombomys opimus*, 9.52% (4/42) of *Meriones libycus*, 4.76% (7/147) of *Niviventer niviventer*, 4.55% (1/22) of *Niviventer confucianus*, 3.57% (1/28) of *Cricetulus longicaudatus*, 2.70% (2/74) of *Myodes rufocanus*, 1.39% (1/72) of *Apodemus draco*, 1.10% (2/181) of *Meriones unguiculatus*, 0.41% (1/244) of *Apodemus agrarius*, 0.24% (1/423) of *Rattus tanezumi*, and 0.22% (1/457) of *Mus musculus* (Table S5). The positive detection was obtained in two eco-climate regions, i.e. Inner Mongolia-Xinjiang (3.64%) and Northern China (1.01%) (Table S5).

Based on *rrs, flaB, and glpQ* genes, RF *Borrelia* genospecies were determined from ticks, with *B. miyamotoi* most frequently seen, taking 44.14% of the positive detection (Figure 1B), which was obtained in a wide region in Northeastern China, Inner Mongolia-Xinjiang, and Northern China. For the first time in China, we determined the presence of *Borrelia theileri* in *R. microplus* and *I. ovatus*, which had 99% identity to *B. theileri* detected in cattle from Zambia (GenBank: LC656239)*.* The unclassified *Borrelia* sp. which were related to *B. theileri* and clustered with the *Borrelia* species detected in Japanese *H. japonica* (GenBank: AB897891) in a separate lineage was determined (Figure 1C-E). The unclassified *Borrelia* sp. was identified in *H. concinna* (*n* = 48), *H. longicornis* (*n* = 6)*, I. persulcatus* (*n* = 4)*, H. japonica* (*n* = 2)*,* and *D. silvarum* (*n* = 2) in Inner Mongolia-Xinjiang, Northeastern and Northern China, respectively.

In the 40 positive wild small mammals, the main species of RF *Borrelia* found was *B. persica* (57.50%), followed by the unclassified *Borrelia* sp*.* that resembled the one detected from the ticks (40.00%), *B. miyamotoi* (2.50%) (Figure 1B). We detected *B. miyamotoi* from 0.41% (1/244) of the *A. agrarius*, representing its ﬁrst identification in rodents. We detected *Borrelia persica* in *R. opimus* (25.68%, 19/74) and *M. libycus* (9.52%, 4/42) in China for the first time*.* The positive detection for the unclassified *Borrelia* sp*.* was obtained from 8 species (*N. niviventer*, *A. draco*, *C. longicaudatus*, *M. unguiculatus*, *M. musculus*, *R. tanezumi*, *M. rufocanus*, *N. confucianus*) of 2 families (*Muridae* and *Cricetidae*) of Rodentia order which were sampled from Inner Mongolia-Xinjiang and Northern China (Table S5).

Here we demonstrated a wide distribution and high variety of RF *Borrelia* in China. Notably two of them, *B. miyamotoi* and *B. persica* were human-pathogenic RF *Borrelia* species. Except for *B. miyamotoi*, another known *Borrelia* species, *B. theileri*, was identified in ticks. We also determined unclassified *Borrelia* sp. which were widely distributed among ticks in three eco-climate regions in China.

Historically, the RF *Borrelia* was considered to follow a cospeciation hypothesis that indicated only one RF *Borrelia* species could be found in a particular host and vector in a given geographic area [14]. Here we demonstrated coexistence of RF *Borrelia* species in one tick species in a given region, with the most frequent coexistence as *B. miyamotoi* and the unclassified *Borrelia* sp. in *D. silvarum, H. japonica, H. longicornis, and I. persulcatus* respectively (Figure 1F, Table S6), all suggesting that the previous geographical distribution studies were not comprehensive.

So far, the reservoir hosts for RF *Borrelia* remained rarely investigated, except for the molecular evidence in rodents and birds in Netherlands [15], bat in China [5]. Our study demonstrated a high diversity of RF *Borrelia* in wild small mammals, yet with only low prevalence in most of the species. Unlike ticks, we determined no coexistence of RF *Borrelia* species in one single mammal species (Figure 1G, Table S7). Their role in the transmission of RF *Borrelia* warrants further investigation.

The study was subject to one major limitation that no isolation of RF *Borrelia* species was achieved, due to their fastidious nature and specialized growth requirement. Recently, multilocus sequence analysis (MLSA) has been accepted as a taxonomic instrument for bacterial species assignment, based on which *Borrelia bavariensis* was proposed as an independent species [16,17]. The species of the unclassified *Borrelia* sp. identified in our study warrants future species classification using isolation and MLSA as previously described for *B. miyamotoi* [18–20]. On the other hand, the current study was mainly focused on the epidemiological investigation, disclosing a wide distribution and high variety of RF *Borrelia* in both tick and wild small mammals with wide distribution in China. We believe the current findings might be valuable for the early warning and prevention of relapsing fever disease, not only in China but also in countries where similar ecological habitat was harboured.

## Supplementary Material

Supplemental Material
