# Supplementary material for: Molecular detection and identification of relapsing fever Borrelia in ticks and wild small mammals in China
Source: Emerg Microbes Infect. 2022 Nov 4;11(1):2632–5. doi: 10.1080/22221751.2022.2134054 (PMC9639508; doi:10.1080/22221751.2022.2134054)
Supplement: Supplemental Material [file TEMI_A_2134054_SM0997.docx]

**Table S1**. **Primers for identifying species of ticks and wild small mammals.**

| **Primer** | **Sequence (5’-3’)** | **Size (bp)** | **Target Gene** | **Reference** |
| --- | --- | --- | --- | --- |
| ixodids-tick-F | CCGGTCTGAACTCAGATCAAGT | 460 | mitochondrial 16S rDNA | (1) |
| ixodids-tick-R | GCTCAATGATTTTTTAAATTGCTGT | 460 | mitochondrial 16S rDNA |  |
| RGlu2L | CAGCATTTAACTGTGACTAATGAC | 762 | cytochrome b | (2) |
| RCb9H | TACACCTAGGAGGTCTTTAATTG | 762 | cytochrome b |  |

**Table S2**. **Primers for detection of relapsing fever *Borrelia.***

| Target gene | Primer | Sequence (5’-3’) | Size (bp) | Reference |
| --- | --- | --- | --- | --- |
| *rrs* | Brm1 | CGCTGTAAACGATGCACACTTGGTGTTAATC | 353 | (3) |
|  | Brm2 | CGGCAGTCTCGTCTGAGTCCCCATCT |  |  |
|  | rrs0F | AAAATAACGAAGAGTTTGATCCTGG | 1533 | (4, 5) |
|  | rrs1533R | GTGATCCAGCCACACTTTCCAGTA |  |  |
|  | Brm1R | GATTAACACCAAGTGTGCATCGTTTACAGCG |  |  |
|  | Brm2F | AGATGGGGACTCAGACGAGACTGCCG |  |  |
| flaB | flaB120F | AGAATTAATMGHGCWTCTGATGATG | 506 | (3, 4) |
|  | flaB764R | GCATCTTCGATCTTTGAAAGTGACATATT |  |  |
|  | flaB625R | CTGGAGCTGCTTGAGCACCTTCT |  |  |
|  | Bor1 | TAATACGTCAGCCATAAATGC | 753 | (6) |
|  | Bor2 | GCTCTTTGATCAGTTATCATTC |  |  |
| *glpQ* | glpQ67F | ATGGGTTCAAACAAAAAGTCACC | 395 | (5) |
|  | glpQ546R | GATGTCTTTACCTTGTTGTTTATGCCA |  |  |
|  | glpQ789R | CCAGGGTCCAATTCCATCAGAATATT |  |  |
|  | glpQ546R | GATGTCTTTACCTTGTTGTTTATGCCA | 480 | (5, 7) |
|  | glpQ-F | CATACGCTTATGCYTTRGGMGCTGA |  |  |
|  | Bp-glpQF | ATAGCTCACAGAGGTGCAAGCGGATATTTACCAGAAC | 810 | (8) |
|  | Bp-glpQR | ATCTTTTACATATGAAGGCAATGCATCAATTCTAAA |  |  |

**Table S3. GenBank accession number for sequences generated in this study.**

| ***Borrelia*** species | **ID** | **Accession numbers** | **Gene** | **Sequence (5'-3')** |
| --- | --- | --- | --- | --- |
| *Borrelia theileri* | HN1F2 | ON059648 | *rrs* | AAAATAACGAAGAGTTTGATCCTGGCTTAGAACTAACGCTGGCAGTGCGTCTTAAGCATGCAAGTCAAACGGAATGTAGCAATACATTCAGTGGCGAACGGGTGAGTAACGCGTGGATAATCTACCTACGAGATGGGGATAACTATTAGAAATAGTAGCTAATACCGAATAAAGTCAATTGAGATGTTAATTGATGAAAGGAAGCCTTTAAAGCTTCGCTTGTAAATGAGTCTGCGTCTTATTAGCTAGTTGGTAGGGTAAGAGCCTACCAAGGCTATGATAAGTAACCGGCCTGAGAGGGTGATCGGTCACACTGGAACTGAGATACGGTCCAGACTCCTACGGGAGGCAGCAGCTAAGAATCTTCCGCAATGGGCGAAAGCCTGACGGAGCGACACTGCGTGAACGAAGAAGGTCGAAAGATTGTAAAGTTCTTTTATAAATGAGGAATAAGCTTTGTAGGAAATGACTAAGTGATGACGTTAGTTTATGAATAAGCCCCGGCTAATTACGTGCCAGCAGCCGCGGTAATACGTAAGGGGCGAGCGTTGTTCGGGATCATTGGGCGTAAAGGGTGAGTAGGCGGATATGTAAGTCTATGTGTAAAATACCACAGCTCAACTGTGGAACTATGCTAGAAACTGCATGACTAGAGTCTGATAGGGGAAGTTAGAATTCCTGGTGTAAGGGTGGAATCTGTTGATATCAGGAAGAATACCAGAGGCGAAGGCGAACTTCTAGGTCAAGACTGACGCTGAGTCACGAAAGCGTAGGGAGCAAACAGGATTAGATACCCTGGTAGTCTACGCTGTAAACGATGCACACTTGGTGTTAATCGAAAGGTTAGTACCGAAGCTAACGTGTTAAGTGTGCCGCCTGGGGAGTATGCTCGCAAGAGTGAAACTCAAAGGAATTGACGGGGGCCCGCACAAGCGGTGGAGCATGTGGTTTAATTCGATGATACGCGAGGAACCTTACCAGGGCTTGACATATACAGGATATAGTTAGAGATAACTACTCCCCTTTTGGGGTCTGTATACAGGTGCTGCATGGTTGTCGTCAGCTCGTGCTGTGAGGTGTTGGGTTAAGTCCCGCAACGAGCGCAACCCTTGTTGTCTGTTACCAGCATGTAAAGATGGGGACTCAGACGAGACTGCCGGTGATAAGCCGGAGGAAGGTGAGGATGACGTCAAATCATCATGGCCCTTATGTCCTGGGCTACACACGTGCTACAATGGCCTGTACAAAGCGATGCGAAACAGTGATGTGAAGCAAAACGCAGAAAGCAGGTCTCAGTCCAGATTGAAGTCTGAAACTCGACTTCATGAAGTTGGAATCGCTAGTAATCGTATATCAGAATGATACGGTGAATACGTTCTCGGGCCTTGTACACACCGCCCGTCACACCACCCGAGTTGAGGATACCCGAAGCTATTATTCTAACCCGCAAGGGAGGAAGGTATCTAAGGTATGTTTAGTGAGGGGGGTGAAGTCGTAACAAGGTAGCCGTACTGGAAAGTGTGGCTGGATCAC |
| *Borrelia theileri* | GD4C9 | ON059649 | *rrs* | AAAATAACGAAGAGTTTGATCCTGGCTTAGAACTAACGCTGGCAGTGCGTCTTAAGCATGCAAGTCAAACGGAATGTAGCAATACATTCAGTGGCGAACGGGTGAGTAACGCGTGGATAATCTACCTACGAGATGGGGATAACTATTAGAAATAGTAGCTAATACCGAATAAAGTCAATTGAGATGTTAATTGATGAAAGGAAGCCTTTAAAGCTTCGCTTGTAAATGAGTCTGCGTCTTATTAGCTAGTTGGTAGGGTAAGAGCCTACCAAGGCTATGATAAGTAACCGGCCTGAGAGGGTGATCGGTCACACTGGAACTGAGATACGGTCCAGACTCCTACGGGAGGCAGCAGCTAAGAATCTTCCGCAATGGGCGAAAGCCTGACGGAGCGACACTGCGTGAACGAAGAAGGTCGAAAGATTGTAAAGTTCTTTTATAAATGAGGAATAAGCTTTGTAGGAAATGACTAAGTGATGACGTTAGTTTATGAATAAGCCCCGGCTAATTACGTGCCAGCAGCCGCGGTAATACGTAAGGGGCGAGCGTTGTTCGGGATCATTGGGCGTAAAGGGTGAGTAGGCGGATATGTAAGTCTATGTGTAAAATACCACAGCTCAACTGTGGAACTATGCTAGAAACTGCATGACTAGAGTCTGATAGGGGAAGTTAGAATTCCTGGTGTAAGGGTGGAATCTGTTGATATCAGGAAGAATACCAGAGGCGAAGGCGAACTTCTAGGTCAAGACTGACGCTGAGTCACGAAAGCGTAGGGAGCAAACAGGATTAGATACCCTGGTAGTCTACGCTGTAAACGATGCACACTTGGTGTTAATCGAAAGGTTAGTACCGAAGCTAACGTGTTAAGTGTGCCGCCTGGGGAGTATGCTCGCAAGAGTGAAACTCAAAGGAATTGACGGGGGCCCGCACAAGCGGTGGAGCATGTGGTTTAATTCGATGATACGCGAGGAACCTTACCAGGGCTTGACATATACAGGATATAGTTAGAGATAACTACTCCCCTTTTGGGGTCTGTATACAGGTGCTGCATGGTTGTCGTCAGCTCGTGCTGTGAGGTGTTGGGTTAAGTCCCGCAACGAGCGCAACCCTTGTTGTCTGTTACCAGCATGTAAAGATGGGGACTCAGACGAGACTGCCGGTGATAAGCCGGAGGAAGGTGAGGATGACGTCAAATCATCATGGCCCTTATGTCCTGGGCTACACACGTGCTACAATGGCCTGTACAAAGCGATGCGAAACAGTGATGTGAAGCAAAACGCAGAAAGCAGGTCTCAGTCCAGATTGAAGTCTGAAACTCGACTTCATGAAGTTGGAATCGCTAGTAATCGTATATCAGAATGATACGGTGAATACGTTCTCGGGCCTTGTACACACCGCCCGTCACACCACCCGAGTTGAGGATACCCGAAGCTATTATTCTAACCCGCAAGGGAGGAAGGTATCTAAGGTATGTTTAGTGAGGGGGGTGAAGTCGTAACAAGGTAGCCGTACTGGAAAGTGTGGCTGGATCAC |
| *Borrelia miyamotoi* | SD6B1 | ON059650 | *rrs* | AAAATAACGAAGAGTTTGATCCTGGCTTAGAACTAACGCTGGCAGTGCGTCTTAAGCATGCAAGTCAGACGGAATGTAGTAATACATTCAGTGGCGAACGGGTGAGTAACGCGTGGATAATCTACCTACGAGATGGGGATAACTATTAGAAATAGTAGCTAATACCGAATAAAGTCAATTAAGATGTTAGTTGATGAAAGGAAGCCTTTAAAGCTTCGCTTGTAGATGAGTCTGCGTCTTATTAGCTAGTTGGTGGGATAAGAGCCTACCAAGGCTATGATAAGTAACCGGCCTGAGAGGGTGATCGGTCACACTGGAACTGAGATACGGTCCAGACTCCTACGGGAGGCAGCAGCTAAGAATCTTCCGCAATGGGCGAAAGCCTGACGGAGCGACACTGCGTGAACGAAGAAGGTCGAAAGATTGTAAAGTTCTTTTATAAGTGAGGAATAAGCTTTGTAGGAAATGACAAAGCGATGACGTTAATTTATGAATAAGCCCCGGCTAATTACGTGCCAGCAGCCGCGGTAATACGTAAGGGGCGAGCGTTGTTCGGGATCATTGGGCGTAAAGGGTGAGTAGGCGGATATGCAAGTCTATGTGTAAAATACCACAGCTCAACTGTGGAACTATGCTGGAAACTGCATGACTAGAGTCTGATAGGGGAAGTTAGAATTCCTGGTGTAAGGGTGGAATCTGTTGATATCAGGAAGAATACCAGAGGCGAAGGCGAACTTCTAGGTCAAGACTGACGCTGAGTCACGAAAGCGTAGGGAGCAAACAGGATTAGATACCCTGGTAGTCTACGCTGTAAACGATGCACACTTGGTGTTAATCGAAAGGTTAGTACCGAAGCTAACGTGTTAAGTGTGCCGCCTGGGGAGTATGTTCGCAAGAATGAAACTCAAAGGAATTGACGGGGGCCCGCACAAGCGGTGGAGCATGTGGTTTAATTCGATGATACGCGAGGAACCTTACCAGGGCTTGACATATACAGGATATAGTTAGAGATAACTATTCCCCGTTTGGGGTCTGTATACAGGTGCTGCATGGTTGTCGTCAGCTCGTGCCGTGAGGTGTTGGGTTAAGTCCCGCAACGAGCGCAACCCTTGTTGTCTGTTACCAGCATGTAAAGATGGGGACTCAGACGAGACTGCCGGTGATAAGCCGGAGGAAGGTGAGGATGACGTCAAATCATCATGGCCCTTATGTCCTGGGCTACACACGTGCTACAATGGCCTGTACAAAGCGATGCGAAACAGTGATGTGAAGCAAAACGCAGAAAGCAGGTCTCAGTCCAGATTGAAGTCTGAAACTCGGCTTCATGAAGTTGGAATCGCTAGTAATCGTATATCAGAATGATACGGTGAATACGTTCTCGGGCCTTGTACACACCGCCCGTCACACCACCCGAGTTGAAGATACCCGAAGCTATTATTCTAACCCGCAAGGGAGGAAGGTATCTAAGGTATGTTTAGTGAGGGGGGTGAAGTCGTAACAAGGTAGCCGTACTGGAAAGTGTGGCTGGATCAC |
| *Borrelia miyamotoi* | HLJ6F5 | ON059651 | *rrs* | AAAATAACGAAGAGTTTGATCCTGGCTTAGAACTAACGCTGGCAGTGCGTCTTAAGCATGCAAGTCAGACGGAATGTAGTAATACATTCAGTGGCGAACGGGTGAGTAACGCGTGGATAATCTACCTACGAGATGGGGATAACTATTAGAAATAGTAGCTAATACCGAATAAAGTCAATTAAGATGTTAGTTGATGAAAGGAAGCCTTTAAAGCTTCGCTTGTAGATGAGTCTGCGTCTTATTAGCTAGTTGGTGGGATAAGAGCCTACCAAGGCTATGATAAGTAACCGGCCTGAGAGGGTGATCGGTCACACTGGAACTGAGATACGGTCCAGACTCCTACGGGAGGCAGCAGCTAAGAATCTTCCGCAATGGGCGAAAGCCTGACGGAGCGACACTGCGTGAACGAAGAAGGTCGAAAGATTGTAAAGTTCTTTTATAAGTGAGGAATAAGCTTTGTAGGAAATGACAAAGCGATGACGTTAATTTATGAATAAGCCCCGGCTAATTACGTGCCAGCAGCCGCGGTAATACGTAAGGGGCGAGCGTTGTTCGGGATCATTGGGCGTAAAGGGTGAGTAGGCGGATATGCAAGTCTATGTGTAAAATACCACAGCTCAACTGTGGAACTATGCTGGAAACTGCATGACTAGAGTCTGATAGGGGAAGTTAGAATTCCTGGTGTAAGGGTGGAATCTGTTGATATCAGGAAGAATACCAGAGGCGAAGGCGAACTTCTAGGTCAAGACTGACGCTGAGTCACGAAAGCGTAGGGAGCAAACAGGATTAGATACCCTGGTAGTCTACGCTGTAAACGATGCACACTTGGTGTTAATCGAAAGGTTAGTACCGAAGCTAACGTGTTAAGTGTGCCGCCTGGGGAGTATGTTCGCAAGAATGAAACTCAAAGGAATTGACGGGGGCCCGCACAAGCGGTGGAGCATGTGGTTTAATTCGATGATACGCGAGGAACCTTACCAGGGCTTGACATATACAGGATATAGTTAGAGATAACTATTCCCCGTTTGGGGTCTGTATACAGGTGCTGCATGGTTGTCGTCAGCTCGTGCCGTGAGGTGTTGGGTTAAGTCCCGCAACGAGCGCAACCCTTGTTGTCTGTTACCAGCATGTAAAGATGGGGACTCAGACGAGACTGCCGGTGATAAGCCGGAGGAAGGTGAGGATGACGTCAAATCATCATGGCCCTTATGTCCTGGGCTACACACGTGCTACAATGGCCTGTACAAAGCGATGCGAAACAGTGATGTGAAGCAAAACGCAGAAAGCAGGTCTCAGTCCAGATTGAAGTCTGAAACTCGGCTTCATGAAGTTGGAATCGCTAGTAATCGTATATCAGAATGATACGGTGAATACGTTCTCGGGCCTTGTACACACCGCCCGTCACACCACCCGAGTTGAAGATACCCGAAGCTATTATTCTAACCCGCAAGGGAGGAAGGTATCTAAGGTATGTTTAGTGAGGGGGGTGAAGTCGTAACAAGGTAGCCGTACTGGAAAGTGTGGCTGGATCAC |
| *Borrelia* sp. | NMG2D7 | ON059652 | *rrs* | AAAATAACGAAGAGTTTGATCCTGGCTTAGAACTAACGCTGGCAGTGCGTCTTAAGCATGCAAGTCAAACGGAATGTAGCAATACATTCAGTGGCGAACGGGTGAGTAACGCGTGGATAATCTACCTACGAGATGGGGATAACTATTAGAAATAGTAGCTAATACCGAATAAAGTCAATTGAGGTGTCAATTGATGAAATGAAGCCTTTAAAGCTTCGCTTGTAGATGAGTCTGCGTCTTATTAGCTAGTTGGTAGGGTAAGAGCCTACCAAGGCTATGATAAGTAACCGGCCTGAGAGGGTGATCGGTCACACTGGAACTGAGATACGGTCCAGACTCCTACGGGAGGCAGCAGCTAAGAATCTTCCGCAATGGGCGAAAGCCTGACGGAGCGACACTGCGTGAACGAAGAAGGTCGAAAGATTGTAAAGTTCTTTTATAAATGAGGAATAAGCTTTGTAGGAAATGACAAAGTGATGACGTTAGTTTATGAATAAGCCCCGGCTAATTACGTGCCAGCAGCCGCGGTAATACGTAAGGGGCGAGCGTTGTTCGGGATCATTGGGCGTAAAGGGTGAGTAGGCGGATATGTAAGTCTATGTGTAAAATACCACAGCTCAACTGTGGAACTATGCTAGAAACTGCATGACTAGAGTCTGATAGGGGAAGTTAGAATTCCTGGTGTAAGGGTGGAATCTGTTGATATCAGGAAGAATACCAGAGGCGAAGGCGAACTTCTAGGTCAAGACTGACGCTGAGTCACGAAAGCGTAGGGAGCAAACAGGATTAGATACCCTGGTAGTCTACGCTGTAAACGATGCACACTGGGTGTTAATCGAAAGATTAGTACCGAAGCTAACGTGTTAAGTGTGCCGCCTGGGGAGTATGCTCGCAAGAGTGAAACTCAAAGGAATTGACGGGGGCCCGCACAAGCGGTGGAGCATGTGGTTTAATTCGATGATACGCGAGGAACCTTACCAGGGCTTGACATATACAGGATATAGTTAGAGATAACTACTCCCCGTTTGGGGTCTGTATACAGGTGCTGCATGGTTGTCGTCAGCTCGTGCTGTGAGGTGTTGGGTTAAGTCCCGCAACGAGCGCAACCCTTATTGTCTGTTACCAGCATGTAAAGATGGGGACTCAGACGAGACTGCCGGTGATAAGCCGGAGGAAGGTGAGGATGACGTCAAATCATCATGGCCCTTATGTCCTGGGCTACACACGTGCTACAATGGCCTGTACAAAGCGATGCGAAACAGTGATGTGAAGCAAAACGCAGAAAGCAGGTCTCAGTCCAGATTGAAGTCTGAAACTCGACTTCATGAAGTTGGAATCGCTAGTAATCGTATATCAGAATGATACGGTGAATACGTTCTCGGGCCTTGTACACACCGCCCGTCACACCACCCGAGTTGAGGATACCCGAAGCTATTATTCTAACCCGCAAGGGAGGAAGGTATCTAAGGTATGTTTAGTGAGGGGGGTGAAGTCGTAACAAGGTAGCCGTACTGGAAAGTGTGGCTGGATCAC |
| *Borrelia* sp. | NMG9B3 | ON059653 | *rrs* | AAAATAACGAAGAGTTTGATCCTGGCTTAGAACTAACGCTGGCAGTGCGTCTTAAGCATGCAAGTCAAACGGAATGTAGCAATACATTCAGTGGCGAACGGGTGAGTAACGCGTGGATAATCTACCTACGAGATGGGGATAACTATTAGAAATAGTAGCTAATACCGAATAAAGTCAATTGAGGTGTCAATTGATGAAATGAAGCCTTTAAAGCTTCGCTTGTAGATGAGTCTGCGTCTTATTAGCTAGTTGGTAGGGTAAGAGCCTACCAAGGCTATGATAAGTAACCGGCCTGAGAGGGTGATCGGTCACACTGGAACTGAGATACGGTCCAGACTCCTACGGGAGGCAGCAGCTAAGAATCTTCCGCAATGGGCGAAAGCCTGACGGAGCGACACTGCGTGAACGAAGAAGGTCGAAAGATTGTAAAGTTCTTTTATAAATGAGGAATAAGCTTTGTAGGAAATGACAAAGTGATGACGTTAGTTTATGAATAAGCCCCGGCTAATTACGTGCCAGCAGCCGCGGTAATACGTAAGGGGCGAGCGTTGTTCGGGATCATTGGGCGTAAAGGGTGAGTAGGCGGATATGTAAGTCTATGTGTAAAATACCACAGCTCAACTGTGGAACTATGCTAGAAACTGCATGACTAGAGTCTGATAGGGGAAGTTAGAATTCCTGGTGTAAGGGTGGAATCTGTTGATATCAGGAAGAATACCAGAGGCGAAGGCGAACTTCTAGGTCAAGACTGACGCTGAGTCACGAAAGCGTAGGGAGCAAACAGGATTAGATACCCTGGTAGTCTACGCTGTAAACGATGCACACTTGGTGTTAATCGAAAGATTAGTACCGAAGCTAACGTGTTAAGTGTGCCGCCTGGGGAGTATGCTCGCAAGAGTGAAACTCAAAGGAATTGACGGGGGCCCGCACAAGCGGTGGAGCATGTGGTTTAATTCGATGATACGCGAGGAACCTTACCAGGGCTTGACATATACAGGATATAGTTAGAGATAACTACTCCCCGTTTGGGGTCTGTATACAGGTGCTGCATGGTTGTCGTCAGCTCGTGCTGTGAGGTGTTGGGTTAAGTCCCGCAACGAGCGCAACCCTTATTGTCTGTTACCAGCATGTAAAGATGGGGACTCAGACGAGACTGCCGGTGATAAGCCGGAGGAAGGTGAGGATGACGTCAAATCATCATGGCCCTTATGTCCTGGGCTACACACGTGCTACAATGGCCTGTACAAAGCGATGCGAAACAGTGATGTGAAGCAAAACGCAGAAAGCAGGTCTCAGTCCAGATTGAAGTCTGAAACTCGACTTCATGAAGTTGGAATCGCTAGTAATCGTATATCAGAATGATACGGTGAATACGTTCTCGGGCCTTGTACACACCGCCCGTCACACCACCCGAGTTGAGGATACCCGAAGCTATTATTCTAACCCGCAAGGGAGGAAGGTATCTAAGGTATGTTTAGTGAGGGGGGTGAAGTCGTAACAAGGTAGCCGTACTGGAAAGTGTGGCTGGATCAC |
| *Borrelia* sp. | NMG9D11 | ON059654 | *rrs* | AAAATAACGAAGAGTTTGATCCTGGCTTAGAACTAACGCTGGCAGTGCGTCTTAAGCATGCAAGTCAAACGGAATGTAGCAATACATTCAGTGACGAACGGGTGAGTAACGCGTGGATAATCTACCTACGAGATGGGGATAACTATTAGAAATAGTAGCTAATACCGAATAAAGTCAATTGAGGTGTCAATTGATGAAATGAAGCCTTTAAAGCTTCGCTTGTAGATGAGTCTGCGTCTTATTAGCTAGTTGGTAGGGTAAGAGCCTACCAAGGCTATGATAAGTAACCGGCCTGAGAGGGTGATCGGTCACACTGGAACTGAGATACGGTCCAGACTCCTACGGGAGGCAGCAGCTAAGAATCTTCCGCAATGGGCGAAAGCCTGACGGAGCGACACTGCGTGAACGAAGAAGGTCGAAAGATTGTAAAGTTCTTTTATAAATGAGGAATAAGCTTTGTAGGAAATGACAAAGTGATGACGTTAGTTTATGAATAAGCCCCGGCTAATTACGTGCCAGCAGCCGCGGTAATACGTAAGGGGCGAGCGTTGTTCGGGATCATTGGGCGTAAAGGGTGAGTAGGCGGATATGTAAGTCTATGTGTAAAATACCACAGCTCAACTGTGGAACTATGCTAGAAACTGCATGACTAGAGTCTGATAGGGGAAGTTAGAATTCCTGGTGTAAGGGTGGAATCTGTTGATATCAGGAAGAATACCAGAGGCGAAGGCGAACTTCTAGGTCAAGACTGACGCTGAGTCACGAAAGCGTAGGGAGCAAACAGGATTAGATACCCTGGTAGTCTACGCTGTAAACGATGCACACTTGGTGTTAATCGAAAGATTAGTACCGAAGCTAACGTGTTAAGTGTGCCGCCTGGGGAGTATGCTCGCAAGAGTGAAACTCAAAGGAATTGACGGGGGCCCGCACAAGCGGTGGAGCATGTGGTTTAATTCGATGATACGCGAGGAACCTTACCAGGGCTTGACATATACAGGATATAGTTAGAGATAACTACTCCCCGTTTGGGGTCTGTATACAGGTGCTGCATGGTTGTCGTCAGCTCGTGCTGTGAGGTGTTGGGTTAAGTCCCGCAACGAGCGCAACCCTTATTGTCTGTTACCAGCATGTAAAGATGGGGACTCAGACGAGACTGCCGGTGATAAGCCGGAGGAAGGTGAGGATGACGTCAAATCATCATGGCCCTTATGTCCTGGGCTACACACGTGCTACAATGGCCTGTACAAAGCGATGCGAAACAGTGATGTGAAGCAAAACGCAGAAAGCAGGTCTCAGTCCAGATTGAAGTCTGAAACTCGACTTCATGAAGTTGGAATCGCTAGTAATCGTATATCAGAATGATACGGTGAATACGTTCTCGGGCCTTGTACACACCGCCCGTCACACCACCCGAGTTGAGGATACCCGAAGCTATTATTCTAACCCGCAAGGGAGGAAGGTATCTAAGGTATGTTTAGTGAGGGGGGTGAAGTCGTAACAAGGTAGCCGTACTGGAAAGTGTGGCTGGATCAC |
| *Borrelia* sp. | NMG10D10 | ON059655 | *rrs* | AAAATAACGAAGAGTTTGATCCTGGCTTAGAACTAACGCTGGCAGTGCGTCTTAAGCATGCAAGTCAAACGGAATGTAGCAATACATTCAGTGGCGAACGGGTGAGTAACGCGTGGATAATCTACCTACGAGATGGGGATAACTATTAGAAATAGTAGCTAATACCGAATAAAGTCAATTGAGGTGTCAATTGATGAAATGAAGCCTTTAAAGCTTCGCTTGTAGATGAGTCTGCGTCTTATTAGCTAGTTGGTAGGGTAAGAGCCTACCAAGGCTATGATAAGTAACCGGCCTGAGAGGGTGATCGGTCACACTGGAACTGAGATACGGTCCAGACTCCTACGGGAGGCAGCAGCTAAGAATCTTCCGCAATGGGCGAAAGCCTGACGGAGCGACACTGCGTGAACGAAGAAGGTCGAAAGATTGTAAAGTTCTTTTATAAATGAGGAATAAGCTTTGTAGGAAATGACAAAGTGATGACGTTAGTTTATGAATAAGCCCCGGCTAATTACGTGCCAGCAGCCGCGGTAATACGTAAGGGGCGAGCGTTGTTCGGGATCATTGGGCGTAAAGGGTGAGTAGGCGGATATGTAAGTCTATGTGTAAAATACCACAGCTCAACTGTGGAACTATGCTAGAAACTGCATGACTAGAGTCTGATAGGGGAAGTTAGAATTCCTGGTGTAAGGGTGGAATCTGTTGATATCAGGAAGAATACCAGAGGCGAAGGCGAACTTCTATGTCAAGACTGACGCTGATTCACGAAAGCGTAGGGAGCAAACAGGATTAGATACCCTGGTAGTCTACGCTGTAAACGATGCACACTGGGTGTTAATCGAAAGATTAGTACCGAAGCTAACGTGTTAAGTGTGCCGCCTGGGGAGTATGCTCGCAAGAGTGAAACTCAAAGGAATTGACGGGGGCCCGCACAAGCGGTGGAGCATGTGGTTTAATTCGATGATACGCGAGGAACCTTACCAGGGCTTGACATATACAGGATATAGTTAGAGATAACTACTCCCCGTTTGGGGTCTGTATACAGGTGCTGCATGGTTGTCGTCAGCTCGTGCTGTGAGGTGTTGGGTTAAGTCCCGCAACGAGCGCAACCCTTATTGTCTGTTACCAGCATGTAAAGATGGGGACTCAGACGAGACTGCCGGTGATAAGCCGGAGGAAGGTGAGGATGACGTCAAATCATCATGGCCCTTATGTCCTGGGCTACACACGTGCTACAATGGCCTGTACAAAGCGATGCGAAACAGTGATGTGAAGCAAAACGCAGAAAGCAGGTCTCAGTCCAGATTGAAGTCTGAAACTCGACTTCATGAAGTTGGAATCGCTAGTAATCGTATATCAGAATGATACGGTGAATACGTTCTCGGGCCTTGTACACACCGCCCGTCACACCACCCGAGTTGAGGATACCCGAAGCTATTATTCTAACCCGCAAGGGAGGAAGGTATCTAAGGTATGTTTAGTGAGGGGGGTGAAGTCGTAACAAGGTAGCCGTACTGGAAAGTGTGGCTGGATCAC |
| *Borrelia miyamotoi* | NMG10H11 | ON059656 | *rrs* | AAAATAACGAAGAGTTTGATCCTGGCTTAGAACTAACGCTGGCAGTGCGTCTTAAGCATGCAAGTCAGACGGAATGTAGTAATACATTCAGTGGCGAACGGGTGAGTAACGCGTGGATAATCTACCTACGAGATGGGGATAACTATTAGAAATAGTAGCTAATACCGAATAAAGTCAATTAAGATGTTAGTTGATGAAAGGAAGCCTTTAAAGCTTCGCTTGTAGATGAGTCTGCGTCTTATTAGCTAGTTGGTGGGATAAGAGCCTACCAAGGCTATGATAAGTAACCGGCCTGAGAGGGTGATCGGTCACACTGGAACTGAGATACGGTCCAGACTCCTACGGGAGGCAGCAGCTAAGAATCTTCCGCAATGGGCGAAAGCCTGACGGAGCGACACTGCGTGAACGAAGAAGGTCGAAAGATTGTAAAGTTCTTTTATAAGTGAGGAATAAGCTTTGTAGGAAATGACAAAGCGATGACGTTAATTTATGAATAAGCCCCGGCTAATTACGTGCCAGCAGCCGCGGTAATACGTAAGGGGCGAGCGTTGTTCGGGATCATTGGGCGTAAAGGGTGAGTAGGCGGATATGCAAGTCTATGTGTAAAATACCACAGCTCAACTGTGGAACTATGCTGGAAACTGCATGACTAGAGTCTGATAGGGGAAGTTAGAATTCCTGGTGTAAGGGTGGAATCTGTTGATATCAGGAAGAATACCAGAGGCGAAGGCGAACTTCTAGGTCAAGACTGACGCTGAGTCACGAAAGCGTAGGGAGCAAACAGGATTAGATACCCTGGTAGTCTACGCTGTAAACGATGCACACTTGGTGTTAATCGAAAGGTTAGTACCGAAGCTAACGTGTTAAGTGTGCCGCCTGGGGAGTATGTTCGCAAGAATGAAACTCAAAGGAATTGACGGGGGCCCGCACAAGCGGTGGAGCATGTGGTTTAATTCGATGATACGCGAGGAACCTTACCAGGGCTTGACATATACAGGATATAGTTAGAGATAACTATTCCCCGTTTGGGGTCTGTATACAGGTGCTGCATGGTTGTCGTCAGCTCGTGCCGTGAGGTGTTGGGTTAAGTCCCGCAACGAGCGCAACCCTTGTTGTCTGTTACCAGCATGTAAAGATGGGGACTCAGACGAGACTGCCGGTGATAAGCCGGAGGAAGGTGAGGATGACGTCAAATCATCATGGCCCTTATGTCCTGGGCTACACACGTGCTACAATGGCCTGTACAAAGCGATGCGAAACAGTGATGTGAAGCAAAACGCAGAAAGCAGGTCTCAGTCCAGATTGAAGTCTGAAACTCGGCTTCATGAAGTTGGAATCGCTAGTAATCGTATATCAGAATGATACGGTGAATACGTTCTCGGGCCTTGTACACACCGCCCGTCACACCACCCGAGTTGAAGATACCCGAAGCTATTATTCTAACCCGCAAGGGAGGAAGGTATCTAAGGTATGTTTAGTGAGGGGGGTGAAGTCGTAACAAGGTAGCCGTACTGGAAAGTGTGGCTGGATCAC |
| *Borrelia theileri* | YN1B2 | ON059657 | *rrs* | AAAATAACGAAGAGTTTGATCCTGGCTTAGAACTAACGCTGGCAGTGCGTCTTAAGCATGCAAGTCAAACGGAATGTAGCAATACATTCAGTGGCGAACGGGTGAGTAACGCGTGGATAATCTACCTACGAGATGGGGATAACTATTAGAAATAGTAGCTAATACCGAATAAAGTCAATTGAGATGTTAATTGATGAAAGGAAGCCTTTAAAGCTTCGCTTGTAAATGAGTCTGCGTCTTATTAGCTAGTTGGTAGGGTAAGAGCCTACCAAGGCTATGATAAGTAACCGGCCTGAGAGGGTGATCGGTCACACTGGAACTGAGATACGGTCCAGACTCCTACGGGAGGCAGCAGCTAAGRATCTTCCGCAATGGGCGAAAGCCTGACGGAGCGACACTGCGTGAACGAAGAAGGTCGAAAGATTGTAAAGTTCTTTTATAAATGAGGAATAAGCTTTGTAGGAAATGACAAAGTGATGACGTTAGTTTATGAATAAGCCCCGGCTAATTACGTGCCAGCAGCCGCGGTAATACGTAAGGGGCGAGCGTTGTTCGGGATCATTGGGCGTAAAGGGTGAGTAGGCGGATATGTAAGTCTATGTGTAAAATACCACAGCTCAACTGTGGAACTATGCTAGAAACTGCATGACTAGAGTCTGATAGGGGAAGTTAGAATTCCTGGTGTAAGGGTGGAATCTGTTGATATCAGGAAGAATACCAGAGGCGAAGGCGAACTTCTAGGTCAAGACTGACGCTGAGTCACGAAAGCGTAGGGAGCAAACAGGATTAGATACCCTGGTAGTCTACGCTGTAAACGATGCACACTTGGTGTTAATCGAAAGGTTAGTACCGAAGCTAACGTGTTAAGTGTGCCGCCTGGGGAGTATGCTCGCAAGAGTGAAACTCAAAGGAATTGACGGGGGCCCGCACAAGCGGTGGAGCATGTGGTTTAATTCGATGATACGCGAGGAACCTTACCAGGGCTTGACATATACAGGATATAGTTAGAGATAACTACTCCCCTTTTGGGGTCTGTATACAGGTGCTGCATGGTTGTCGTCAGCTCGTGCTGTGAGGTGTTGGGTTAAGTCCCGCAACGAGCGCAACCCTTGTTGTCTGTTACCAGCATGTAAAGATGGGGACTCAGACGAGACTGCCGGTGATAAGCCGGAGGAAGGTGAGGATGACGTCAAATCATCATGGCCCTTATGTCCTGGGCTACACACGTGCTACAATGGCCTGTACAAAGCGATGCGAAACAGTGATGTGAAGCAAAACGCAGAAAGCAGGTCTCAGTCCAGATTGAAGTCTGAAACTCGACTTCATGAAGTTGGAATCGCTAGTAATCGTATATCAGAATGATACGGTGAATACGTTCTCGGGCCTTGTACACACCGCCCGTCACACCACCCGAGTTGAGGATACCCGAAGCTATTATTCTAACCCGCAAGGGAGGAAGGTATCTAAGGTATGTTTAGTGAGGGGGGTGAAGTCGTAACAAGGTAGCCGTACTGGAAAGTGTGGCTGGATCAC |
| *Borrelia* sp. | HNQW5 | ON059658 | *rrs* | AAAATAACGAAGAGTTTGATCCTGGCTTAGAACTAACGCTGGCAGTGCGTCTTAAGCATGCAAGTCGAACGGGATGTAGCAATACATTCAGTGGCGAACGGGTGAGTAGCGCGTGGATAATCTACCTACGAGATGGGGATAACTACTAGAAATAGTAGCTAATACCGAATAAAGTCAATTGAGATGTCAATTGATGAAAGGAAGCCTTTAAAGCTTCGCTTGTAGATGAGTCTGCGTCTTATTAGCTAGTTGGTAGGGTAAGAGCCTACCAAGGCTATGATAAGTAACCGGCCTGAGAGGGTGATCGGTCACACTGGAACTGAGATACGGTCCAGACTCCTACGGGAGGCAGCAGCTAAGAATCTTCCGCAATGGGCGAAAGCCTGACGGAGCGACACTGCGTGAACGAAGAAGGTCGAAAGATTGTAAAGTTCTTTTATAAATGAGGAATAAGCTTTGTAGGAAATGACAAAGTGATGACGTTAGTTTATGAATAAGCCCCGGCTAATTACGTGCCAGCAGCCGCGGTAATACGTAAGGGGCGAGCGTTGTTCGGGATCATTGGGCGTAAAGGGTGAGTAGGCGGATATGTAAGTCTATGTGTAAAATACCACAGCTCAACTGTGGAACTATGCTAGAAACTGCATGACTAGAGTCTGATAGGGGAAGTTAGAATTCCTGGTGTAAGGGTGGAATCTGTTGATATCAGGAAGAATACCAGAGGCGAAGGCGAACTTCTAGGTCAAGACTGACGCTGAGTCACGAAAGCGTAGGGAGCAAACAGGATTAGATACCCTGGTAGTCTACGCTGTAAACGATGCACACTTGGTGTTAATCGAAAGATTAGTACCGAAGCTAACGTGTTAAGTGTGCCGCCTGGGGAGTATGCTCGCAAGAGTGAAACTCAAAGGAATTGACGGGGGCCCGCACAAGCGGTGGAGCATGTGGTTTAATTCGATGATACGCGAGGAACCTTACCAGGGCTTGACATATACAGGATATAGTTAGAGATAACTACTCCCCGTTTGGGGTCTGTATACAGGTGCTGCATGGTTGTCGTCAGCTCGTGCTGTGAGGTGTTGGGTTAAGTCCCGCAACGAGCGCAACCCTTATTGTCTGTTACCAGCATGTAAAGATGGGGACTCAGACGAGACTGCCGGTGATAAGCCGGAGGAAGGTGAGGATGACGTCAAATCATCATGGCCCTTATGTCCTGGGCTACACACGTGCTACAATGGCCTGTACAAAGCGATGCGAAACAGTGATGTGAAGCAAAACGCAGAAAGCAGGTCTCAGTCCAGATTGAAGTCTGAAACTCGACTTCATGAAGTTGGAATCGCTAGTAATCGTATATCAGAATGATACGGTGAATACGTTCTCGGGCCTTGTACACACCGCCCGTCACACCACCCGAGTTGAGGATACCCGAAGCTATTATTCTAACCCGCAAGGGAGGAAGGTATCTAAGGTATGTTTAGTGAGGGGGGTGAAGTCGTAACAAGGTAGCCGTACTGGAAAGTGTGGCTGGATCAC |
| *Borrelia* sp. | SDJN171 | ON209364 | *rrs* | AAAATAACGAAGAGTTTGATCCTGGCTTAGAACTAACGCTGGCAGTGCGTCTTAAGCATGCAAGTCGAACGGGATGTAGCAATACATTCAGTGGCGAACGGGTGAGTAGCGCGTGGATAATCTACCTACGAGATGGGGATAACTACTAGAAATAGTAGCTAATACCGAATAAAGTCAATTGAGATGTCAATTGATGAAAGGAAGCCTTTAAAGCTTCGCTTGTAGATGAGTCTGCGTCTTATTAGCTAGTTGGTAGGGTAAGAGCCTACCAAGGCTATGATAAGTAACCGGCCTGAGAGGGTGATCGGTCACACTGGAACTGAGATACGGTCCAGACTCCTACGGGAGGCAGCAGCTAAGAATCTTCCGCAATGGGCGAAAGCCTGACGGAGCGACACTGCGTGAACGAAGAAGGTCGAAAGATTGTAAAGTTCTTTTATAAATGAGGAATAAGCTTTGTAGGAAATGACAAAGTGATGACGTTAGTTTATGAATAAGCCCCGGCTAATTACGTGCCAGCAGCCGCGGTAATACGTAAGGGGCGAGCGTTGTTCGGGATCATTGGGCGTAAAGGGTGAGTAGGCGGATATGTAAGTCTATGTGTAAAATACCACAGCTCAACTGTGGAACTATGCTAGAAACTGCATGACTAGAGTCTGATAGGGGAAGTTAGAATTCCTGGTGTAAGGGTGGAATCTGTTGATATCAGGAAGAATACCAGAGGCGAAGGCGAACTTCTAGGTCAAGACTGACGCTGAGTCACGAAAGCGTAGGGAGCAAACAGGATTAGATACCCTGGTAGTCTACGCTGTAAACGATGCACACTTGGTGTTAATCGAAAGATTAGTACCGAAGCTAACGTGTTAAGTGTGCCGCCTGGGGAGTATGCTCGCAAGAGTGAAACTCAAAGGAATTGACGGGGGCCCGCACAAGCGGTGGAGCATGTGGTTTAATTCGATGATACGCGAGGAACCTTACCAGGGCTTGACATATACAGGATATAGTTAGAGATAACTACTCCCCGTTTGGGGTCTGTATACAGGTGCTGCATGGTTGTCGTCAGCTCGTGCTGTGAGGTGTTGGGTTAAGTCCCGCAACGAGCGCAACCCTTATTGTCTGTTACCAGCATGTAAAGATGGGGACTCAGACGAGACTGCCGGTGATAAGCCGGAGGAAGGTGAGGATGACGTCAAATCATCATGGCCCTTATGTCCTGGGCTACACACGTGCTACAATGGCCTGTACAAAGCGATGCGAAACAGTGATGTGAAGCAAAACGCAG-AAAGCAGGTCTCAGTCCAGATTGAAGTCTGAAACTCGACTTCATGAAGTTGGAATCGCTAGTAATCGTATATCAGAATGATACGGTGAATACGTTCTCGGGCCTTGTACACACCGCCCGTCACACCACCCGAGTTGAGGATACCCGAAGCTATTATTCTAACCCGCAAGGGAGGAAGGTATCTAAGGTATGTTTAGTGAGGGGGGTGAAGTCGTAACAAGGTAGCCGTACTGGAAAGTGTGGCTGGATCAC |
| *Borrelia miyamotoi* | HLJ7E3 | ON209365 | *rrs* | AAAATAACGAAGAGTTTGATCCTGGCTTAGAACTAACGCTGGCAGTGCGTCTTAAGCATGCAAGTCAGACGGAATGTAGTAATACATTCAGTGGCGAACGGGTGAGTAACGCGTGGATAATCTACCTACGAGATGGGGATAACTATTAGAAATAGTAGCTAATACCGAATAAAGTCAATTAAGATGTTAGTTGATGAAAGGAAGCCTTTAAAGCTTCGCTTGTAGATGAGTCTGCGTCTTATTAGCTAGTTGGTGGGATAAGAGCCTACCAAGGCTATGATAAGTAACCGGCCTGAGAGGGTGATCGGTCACACTGGAACTGAGATACGGTCCAGACTCCTACGGGAGGCAGCAGCTAAGAATCTTCCGCAATGGGCGAAAGCCTGACGGAGCGACACTGCGTGAACGAAGAAGGTCGAAAGATTGTAAAGTTCTTTTATAAGTGAGGAATAAGCTTTGTAGGAAATGACAAAGCGATGACGTTAATTTATGAATAAGCCCCGGCTAATTACGTGCCAGCAGCCGCGGTAATACGTAAGGGGCGAGCGTTGTTCGGGATCATTGGGCGTAAAGGGTGAGTAGGCGGATATGCAAGTCTATGTGTAAAATACCACAGCTCAACTGTGGAACTATGCTGGAAACTGCATGACTAGAGTCTGATAGGGGAAGTTAGAATTCCTGGTGTAAGGGTGGAATCTGTTGATATCAGGAAGAATACCAGAGGCGAAGGCGAACTTCTAGGTCAAGACTGACGCTGAGTCACGAAAGCGTAGGGAGCAAACAGGATTAGATACCCTGGTAGTCTACGCTGTAAACGATGCACACTTGGTGTTAATCGAAAGGTTAGTACCGAAGCTAACGTGTTAAGTGTGCCGCCTGGGGAGTATGTTCGCAAGAATGAAACTCAAAGGAATTGACGGGGGCCCGCACAAGCGGTGGAGCATGTGGTTTAATTCGATGATACGCGAGGAACCTTACCAGGGCTTGACATATACAGGATATAGTTAGAGATAACTATTCCCCGTTTGGGGTCTGTATACAGGTGCTGCATGGTTGTCGTCAGCTCGTGCCGTGAGGTGTTGGGTTAAGTCCCGCAACGAGCGCAACCCTTGTTGTCTGTTACCAGCATGTAAAGATGGGGACTCAGACGAGACTGCCGGTGATAAGCCGGAGGAAGGTGAGGATGACGTCAAATCATCATGGCCCTTATGTCCTGGGCTACACACGTGCTACAATGGCCTGTACAAAGCGATGCGAAACAGTGATGTGAAGCAAAACGCAG-AAAGCAGGTCTCAGTCCAGATTGAAGTCTGAAACTCGGCTTCATGAAGTTGGAATCGCTAGTAATCGTATATCAGAATGATACGGTGAATACGTTCTCGGGCCTTGTACACACCGCCCGTCACACCACCCGAGTTGAAGATACCCGAAGCTATTATTCTAACCCGCAAGGGAGGAAGGTATCTAAGGTATGTTTAGTGAGGGGGGTGAAGTCGTAACAAGGTAGCCGTACTGGAAAGTGTGGCTGGATCAC |
| *Borrelia* sp. | NMG9F5 | ON209366 | *rrs* | AAAATAACGAAGAGTTTGATCCTGGCTTAGAACTAACGCTGGCAGTGCGTCTTAAGCATGCAAGTCAAACGGAATGTAGCAATACATTCAGTGGCGAACGGGTGAGTAACGCGTGGATAATCTACCTACGAGATGGGGATAACTATTAGAAATAGTAGCTAATACCGAATAAAGTCAATTGAGGTGTCAATTGATGAAATGAAGCCTTTAAAGCTTCGCTTGTAGATGAGTCTGCGTCTTATTAGCTAGTTGGTAGGGTAAGAGCCTACCAAGGCTATGATAAGTAACCGGCCTGAGAGGGTGATCGGTCACACTGGAACTGAGATACGGTCCAGACTCCTACGGGAGGCAGCAGCTAAGAATCTTCCGCAATGGGCGAAAGCCTGACGGAGCGACACTGCGTGAACGAAGAAGGTCGAAAGATTGTAAAGTTCTTTTATAAATGAGGAATAAGCTTTGTAGGAAATGACAAAGTGATGACGTTAGTTTATGAATAAGCCCCGGCTAATTACGTGCCAGCAGCCGCGGTAATACGTAAGGGGCGAGCGTTGTTCGGGATCATTGGGCGTAAAGGGTGAGTAGGCGGATATGTAAGTCTATGTGTAAAATACCACAGCTCAACTGTGGAACTATGCTAGAAACTGCATGACTAGAGTCTGATAGGGGAAGTTAGAATTCCTGGTGTAAGGGTGGAATCTGTTGATATCAGGAAGAATACCAGAGGCGAAGGCGAACTTCTAGGTCAAGACTGACGCTGAGTCACGAAAGCGTAGGGAGCAAACAGGATTAGATACCCTGGTAGTCTACGCTGTAAACGATGCACACTGGGTGTTAATCGAAAGATTAGTACCGAAGCTAACGTGTTAAGTGTGCCGCCTGGGGAGTATGCTCGCAAGAGTGAAACTCAAAGGAATTGACGGGGGCCCGCACAAGCGGTGGAGCATGTGGTTTAATTCGATGATACGCGAGGAACCTTACCAGGGCTTGACATATACAGGATATAGTTAGAGATAACTACTCCCCGTTTGGGGTCTGTATACAGGTGCTGCATGGTTGTCGTCAGCTCGTGCTGTGAGGTGTTGGGTTAAGTCCCGCAACGAGCGCAACCCTTATTGTCTGTTACCAGCATGTAAAGATGGGGACTCAGACGAGACTGCCGGTGATAAGCCGGAGGAAGGTGAGGATGACGTCAAATCATCATGGCCCTTATGTCCTGGGCTACACACGTGCTACAATGGCCTGTACAAAGCGATGCGAAACAGTGATGTGAAGCAAAACGCAG-AAAGCAGGTCTCAGTCCAGATTGAAGTCTGAAACTCGACTTCATGAAGTTGGAATCGCTAGTAATCGTATATCAGAATGATACGGTGAATACGTTCTCGGGCCTTGTACACACCGCCCGTCACACCACCCGAGTTGAGGATACCCGAAGCTATTATTCTAACCCGCAAGGGAGGAAGGTATCTAAGGTATGTTTAGTGAGGGGGGTGAAGTCGTAACAAGGTAGCCGTACTGGAAAGTGTGGCTGGATCAC |
| *Borrelia persica* | XJAL048 | ON209367 | *rrs* | AAAATAACGAAGAGTTTGATCCTGGCTTAGAACTAACGCTGGCAGTGCGTCTTAAGCATGCAAGTCAGACGGGATGTAGCAATACATTCAGTGGCGAACGGGTGAGTAACGCGTGGATAATCTACCTATGAGATGGGGATAACTATTAGAAATAGTAGCTAATACCGAATAAGGTCAGTTGAGATGTCAATTGATGAAAGGAAGCCTTTAAAGCTTCGCTTGTAGATGAGTCTGCGTCTTATTAGCTAGTTGGTGGGGTAAGAGCCTACCAAGGCTATGATAAGTAACCGGCCTGAGAGGGTGAACGGTCACACTGGAACTGAGATACGGTCCAGACTCCTACGGGAGGCAGCAGCTAAGAATCTTCCGCAATGGGCGAAAGCCTGACGGAGCGACACTGCGTGAACGAAGAAGGTCGAAAGATTGTAAAGTTCTTTTATGAATGAGGAATAAGCCTTGTAGGAAATGACAAGGTGATGACGTTAATTTATGAATAAGCCCCGGCTAATTACGTGCCAGCAGCCGCGGTAATACGTAAGGGGCGAGCGTTGTTCGGGATTATTGGGCGTAAAGGGTGAGTAGGCGGATATGCAAGTCTATGCGTAAAATACCACAGCTCAACTGTGGAGCTATGCTGGAAACTGCATGACTAGAGTCTGATAGGGGAAGTTAGAATTCCTGGTGTAAGGGTGGAATCTGTTGATATCAGGAAGAATACCAGAGGCGAAGGCGAACTTCTGGGTCAAGACTGACGCTGAGTCACGAAAGCGTAGGGAGCAAACAGGATTAGATACCCTGGTAGTCTACGCTGTAAACGATGCACACTTGGTGTTAATCGAGAGATTAGTACCGAAGCTAACGTGTTAAGTGTGCCGCCTGGGGAGTATGCTCGCAAGAGTGAAACTCAAAGGAATTGACGGGGGCCCGCACAAGCGGTGGAGCATGTGGTTTAATTCGATGATACGCGAGAAACCTTACCAGGGCTTGACATATACAGGATGTAGTTAGAGATAATTATTCCCCGTTTGGGGTCTGTATACAGGTGCTGCATGGTTGTCGTCAGCTCGTGCTGTGAGGTGTTGGGTTAAGTCCCGCAACGAGCGCAACCCTTATTGTCTGTTACCAGCATGTAAAGATGGGAACTCAGATGAGACTGCCGGTGATAAGCCGGAGGAAGGTGAGGATGACGTCAAATCATCATGGCCCTTATGCCCTGGGCTACACACGTGCTACAATGGCCTGTACAAAGCGAAGCGAAACAGTGATGTGAAGCAAAACGCATTAAAGCAGGTCTCAGTCCAGATTGAAGTCTGAAACTCGACTTCATGAAGTTGGAATCGCTAGTAATCGTATATCAGAATGATACGGTGAATACGTTCTCGGGCCTTGTACACACCGCCCGTCACACCACCCGAGTTGAGGATACCCGAAGCTATTATTCTAACCCGCAAGGGAGGAAGGTATCTAAGGTATGTTTAGTGAGGGGGGTGAAGTCGTAACAAGGTAGCCGTACTGGAAAGTGTGGCTGGATCAC |
| *Borrelia persica* | XJAL009 | ON209368 | *rrs* | AAAATAACGAAGAGTTTGATCCTGGCTTAGAACTAACGCTGGCAGTGCGTCTTAAGCATGCAAGTCAGACGGGATGTAGCAATACATTCAGTGGCGAACGGGTGAGTAACGCGTGGATAATCTACCTATGAGATGGGGATAACTATTAGAAATAGTAGCTAATACCGAATAAGGTCAGTTGAGATGTCAATTGATGAAAGGAAGCCTTTAAAGCTTCGCTTGTAGATGAGTCTGCGTCTTATTAGCTAGTTGGTGGGGTAAGAGCCTACCAAGGCTATGATAAGTAACCGGCCTGAGAGGGTGAACGGTCACACTGGAACTGAGATACGGTCCAGACTCCTACGGGAGGCAGCAGCTAAGAATCTTCCGCAATGGGCGAAAGCCTGACGGAGCGACACTGCGTGAACGAAGAAGGTCGAAAGATTGTAAAGTTCTTTTATGAATGAGGAATAAGCCTTGTAGGAAATGACAAGGTGATGACGTTAATTTATGAATAAGCCCCGGCTAATTACGTGCCAGCAGCCGCGGTAATACGTAAGGGGCGAGCGTTGTTCGGGATTATTGGGCGTAAAGGGTGAGTAGGCGGATATGCAAGTCTATGCGTAAAATACCACAGCTCAACTGTGGAGCTATGCTGGAAACTGCATGACTAGAGTCTGATAGGGGAAGTTAGAATTCCTGGTGTAAGGGTGGAATCTGTTGATATCAGGAAGAATACCAGAGGCGAAGGCGAACTTCTGGGTCAAGACTGACGCTGAGTCACGAAAGCGTAGGGAGCAAACAGGATTAGATACCCTGGTAGTCTACGCTGTAAACGATGCACACTTGGTGTTAATCGAGAGATTAGTACCGAAGCTAACGTGTTAAGTGTGCCGCCTGGGGAGTATGCTCGCAAGAGTGAAACTCAAAGGAATTGACGGGGGCCCGCACAAGCGGTGGAGCATGTGGTTTAATTCGATGATACGCGAGAAACCTTACCAGGGCTTGACATATACAGGATGTAGTTAGAGATAATTATTCCCCGTTTGGGGTCTGTATACAGGTGCTGCATGGTTGTCGTCAGCTCGTGCTGTGAGGTGTTGGGTTAAGTCCCGCAACGAGCGCAACCCTTATTGTCTGTTACCAGCATGTAAAGATGGGAACTCAGATGAGACTGCCGGTGATAAGCCGGAGGAAGGTGAGGATGACGTCAAATCATCATGGCCCTTATGCCCTGGGCTACACACGTGCTACAATGGCCTGTACAAAGCGAAGCGAAACAGTGATGTGAAGCAAAACGCATTAAAGCAGGTCTCAGTCCAGATTGAAGTCTGAAACTCGACTTCATGAAGTTGGAATCGCTAGTAATCGTATATCAGAATGATACGGTGAATACGTTCTCGGGCCTTGTACACACCGCCCGTCACACCACCCGAGTTGAGGATACCCGAAGCTATTATTCTAACCCGCAAGGGAGGAAGGTATCTAAGGTATGTTTAGTGAGGGGGGTGAAGTCGTAACAAGGTAGCCGTACTGGAAAGTGTGGCTGGATCAC |
| *Borrelia persica* | XJDLT014 | ON209369 | *rrs* | AAAATAACGAAGAGTTTGATCCTGGCTTAGAACTAACGCTGGCAGTGCGTCTTAAGCATGCAAGTCAGACGGGATGTAGCAATACATTCAGTGGCGAACGGGTGAGTAACGCGTGGATAATCTACCTATGAGATGGGGATAACTATTAGAAATAGTAGCTAATACCGAATAAGGTCAGTTGAGATGTCAATTGATGAAAGGAAGCCTTTAAAGCTTCGCTTGTAGATGAGTCTGCGTCTTATTAGCTAGTTGGTGGGGTAAGAGCCTACCAAGGCTATGATAAGTAACCGGCCTGAGAGGGTGAACGGTCACACTGGAACTGAGATACGGTCCAGACTCCTACGGGAGGCAGCAGCTAAGAATCTTCCGCAATGGGCGAAAGCCTGACGGAGCGACACTGCGTGAACGAAGAAGGTCGAAAGATTGTAAAGTTCTTTTATGAATGAGGAATAAGCCTTGTAGGAAATGACAAGGTGATGACGTTAATTTATGAATAAGCCCCGGCTAATTACGTGCCAGCAGCCGCGGTAATACGTAAGGGGCGAGCGTTGTTCGGGATTATTGGGCGTAAAGGGTGAGTAGGCGGATATGCAAGTCTATGCGTAAAATACCACAGCTCAACTGTGGAGCTATGCTGGAAACTGCATGACTAGAGTCTGATAGGGGAAGTTAGAATTCCTGGTGTAAGGGTGGAATCTGTTGATATCAGGAAGAATACCAGAGGCGAAGGCGAACTTCTGGGTCAAGACTGACGCTGAGTCACGAAAGCGTAGGGAGCAAACAGGATTAGATACCCTGGTAGTCTACGCTGTAAACGATGCACACTTGGTGTTAATCGAGAGATTAGTACCGAAGCTAACGTGTTAAGTGTGCCGCCTGGGGAGTATGCTCGCAAGAGTGAAACTCAAAGGAATTGACGGGGGCCCGCACAAGCGGTGGAGCATGTGGTTTAATTCGATGATACGCGAGAAACCTTACCAGGGCTTGACATATACAGGATGTAGTTAGAGATAATTATTCCCCGTTTGGGGTCTGTATACAGGTGCTGCATGGTTGTCGTCAGCTCGTGCTGTGAGGTGTTGGGTTAAGTCCCGCAACGAGCGCAACCCTTATTGTCTGTTACCAGCATGTAAAGATGGGAACTCAGATGAGACTGCCGGTGATAAGCCGGAGGAAGGTGAGGATGACGTCAAATCATCATGGCCCTTATGCCCTGGGCTACACACGTGCTACAATGGCCTGTACAAAGCGAAGCGAAACAGTGATGTGAAGCAAAACGCATTAAAGCAGGTCTCAGTCCAGATTGAAGTCTGAAACTCGACTTCATGAAGTTGGAATCGCTAGTAATCGTATATCAGAATGATACGGTGAATACGTTCTCGGGCCTTGTACACACCGCCCGTCACACCACCCGAGTTGAGGATACCCGAAGCTATTATTCTAACCCGCAAGGGAGGAAGGTATCTAAGGTATGTTTAGTGAGGGGGGTGAAGTCGTAACAAGGTAGCCGTACTGGAAAGTGTGGCTGGATCAC |
| *Borrelia persica* | XJDLT021 | ON209370 | *rrs* | AAAATAACGAAGAGTTTGATCCTGGCTTAGAACTAACGCTGGCAGTGCGTCTTAAGCATGCAAGTCAGACGGGATGTAGCAATACATTCAGTGGCGAACGGGTGAGTAACGCGTGGATAATCTACCTATGAGATGGGGATAACTATTAGAAATAGTAGCTAATACCGAATAAGGTCAGTTGAGATGTCAATTGATGAAAGGAAGCCTTTAAAGCTTCGCTTGTAGATGAGTCTGCGTCTTATTAGCTAGTTGGTGGGGTAAGAGCCTACCAAGGCTATGATAAGTAACCGGCCTGAGAGGGTGAACGGTCACACTGGAACTGAGATACGGTCCAGACTCCTACGGGAGGCAGCAGCTAAGAATCTTCCGCAATGGGCGAAAGCCTGACGGAGCGACACTGCGTGAACGAAGAAGGTCGAAAGATTGTAAAGTTCTTTTATGAATGAGGAATAAGCCTTGTAGGAAATGACAAGGTGATGACGTTAATTTATGAATAAGCCCCGGCTAATTACGTGCCAGCAGCCGCGGTAATACGTAAGGGGCGAGCGTTGTTCGGGATTATTGGGCGTAAAGGGTGAGTAGGCGGATATGCAAGTCTATGCGTAAAATACCACAGCTCAACTGTGGAGCTATGCTGGAAACTGCATGACTAGAGTCTGATAGGGGAAGTTAGAATTCCTGGTGTAAGGGTGGAATCTGTTGATATCAGGAAGAATACCAGAGGCGAAGGCGAACTTCTGGGTCAAGACTGACGCTGAGTCACGAAAGCGTAGGGAGCAAACAGGATTAGATACCCTGGTAGTCTACGCTGTAAACGATGCACACTTGGTGTTAATCGAGAGATTAGTACCGAAGCTAACGTGTTAAGTGTGCCGCCTGGGGAGTATGCTCGCAAGAGTGAAACTCAAAGGAATTGACGGGGGCCCGCACAAGCGGTGGAGCATGTGGTTTAATTCGATGATACGCGAGAAACCTTACCAGGGCTTGACATATACAGGATGTAGTTAGAGATAATTATTCCCCGTTTGGGGTCTGTATACAGGTGCTGCATGGTTGTCGTCAGCTCGTGCTGTGAGGTGTTGGGTTAAGTCCCGCAACGAGCGCAACCCTTATTGTCTGTTACCAGCATGTAAAGATGGGAACTCAGATGAGACTGCCGGTGATAAGCCGGAGGAAGGTGAGGATGACGTCAAATCATCATGGCCCTTATGCCCTGGGCTACACACGTGCTACAATGGCCTGTACAAAGCGAAGCGAAACAGTGATGTGAAGCAAAACGCATTAAAGCAGGTCTCAGTCCAGATTGAAGTCTGAAACTCGACTTCATGAAGTTGGAATCGCTAGTAATCGTATATCAGAATGATACGGTGAATACGTTCTCGGGCCTTGTACACACCGCCCGTCACACCACCCGAGTTGAGGATACCCGAAGCTATTATTCTAACCCGCAAGGGAGGAAGGTATCTAAGGTATGTTTAGTGAGGGGGGTGAAGTCGTAACAAGGTAGCCGTACTGGAAAGTGTGGCTGGATCAC |
| *Borrelia persica* | XJAL047 | ON209371 | *rrs* | AAAATAACGAAGAGTTTGATCCTGGCTTAGAACTAACGCTGGCAGTGCGTCTTAAGCATGCAAGTCAGACGGGATGTAGCAATACATTCAGTGGCGAACGGGTGAGTAACGCGTGGATAATCTACCTATGAGATGGGGATAACTATTAGAAATAGTAGCTAATACCGAATAAGGTCAGTTGAGATGTCAATTGATGAAAGGAAGCCTTTAAAGCTTCGCTTGTAGATGAGTCTGCGTCTTATTAGCTAGTTGGTGGGGTAAGAGCCTACCAAGGCTATGATAAGTAACCGGCCTGAGAGGGTGAACGGTCACACTGGAACTGAGATACGGTCCAGACTCCTACGGGAGGCAGCAGCTAAGAATCTTCCGCAATGGGCGAAAGCCTGACGGAGCGACACTGCGTGAACGAAGAAGGTCGAAAGATTGTAAAGTTCTTTTATGAATGAGGAATAAGCCTTGTAGGAAATGACAAGGTGATGACGTTAATTTATGAATAAGCCCCGGCTAATTACGTGCCAGCAGCCGCGGTAATACGTAAGGGGCGAGCGTTGTTCGGGATTATTGGGCGTAAAGGGTGAGTAGGCGGATATGCAAGTCTATGCGTAAAATACCACAGCTCAACTGTGGAGCTATGCTGGAAACTGCATGACTAGAGTCTGATAGGGGAAGTTAGAATTCCTGGTGTAAGGGTGGAATCTGTTGATATCAGGAAGAATACCAGAGGCGAAGGCGAACTTCTGGGTCAAGACTGACGCTGAGTCACGAAAGCGTAGGGAGCAAACAGGATTAGATACCCTGGTAGTCTACGCTGTAAACGATGCACACTTGGTGTTAATCGAGAGATTAGTACCGAAGCTAACGTGTTAAGTGTGCCGCCTGGGGAGTATGCTCGCAAGAGTGAAACTCAAAGGAATTGACGGGGGCCCGCACAAGCGGTGGAGCATGTGGTTTAATTCGATGATACGCGAGAAACCTTACCAGGGCTTGACATATACAGGATGTAGTTAGAGATAATTATTCCCCGTTTGGGGTCTGTATACAGGTGCTGCATGGTTGTCGTCAGCTCGTGCTGTGAGGTGTTGGGTTAAGTCCCGCAACGAGCGCAACCCTTATTGTCTGTTACCAGCATGTAAAGATGGGAACTCAGATGAGACTGCCGGTGATAAGCCGGAGGAAGGTGAGGATGACGTCAAATCATCATGGCCCTTATGCCCTGGGCTACACACGTGCTACAATGGCCTGTACAAAGCGAAGCGAAACAGTGATGTGAAGCAAAACGCATTAAAGCAGGTCTCAGTCCAGATTGAAGTCTGAAACTCGACTTCATGAAGTTGGAATCGCTAGTAATCGTATATCAGAATGATACGGTGAATACGTTCTCGGGCCTTGTACACACCGCCCGTCACACCACCCGAGTTGAGGATACCCGAAGCTATTATTCTAACCCGCAAGGGAGGAAGGTATCTAAGGTATGTTTAGTGAGGGGGGTGAAGTCGTAACAAGGTAGCCGTACTGGAAAGTGTGGCTGGATCAC |
| *Borrelia miyamotoi* | SD4195 | ON365959 | *rrs* | AGTCAGACGGAATGTAGTAATACATTCAGTGGCGAACGGGTGAGTAACGCGTGGATAATCTACCTACGAGATGGGGATAACTATTAGAAATAGTAGCTAATACCGAATAAAGTCAATTGAGGTGTTAGTTGATGAAAGGAAGCCTTTAAAGCTTCGCTTGTAGATGAGTCTGCGTCTTATTAGCTAGTTGGTGGGGTAAGAGCCTACCAAGGCTATGATAAGTAACCGGCCTGAGAGGGTGATCGGTCACACTGGAACTGAGATACGGTCCAGACTCCTACGGGAGGCAGCAGCTAAGAATCTTCCGCAATGGGCGAAAGCCTGACGGAGCGACACTGCGTGAACGAAGAAGGTCGAAAGATTGTAAAGTTCTTTTATAAATGAGGAATAAGCTTTGTAGGAAATGACAAAATGATGACGTTAATTTAGGAATAAGCCCCGGCTAATTACGTGCCAGCAGCCGCGGTAATACGTAAGGGGCGAGCGTTGTTCGGGATCATTGGGCGTAAAGGGTGAGTAGGCGGATATGCAAGTCTATGTGTAAAATACCACAGCTCAACTGTGGAACTATGCTGGAAACTGCATGACTAGAGTCTGATAGGGGAAGTTAGAATTCCTGGTGTAAGGGTGGAATCTGTTGATATCAGGAAGAATACCAGAGGCGAAGGCGAACTTCTAGGTCAAGACTGACGCTGAGTCACGAAAGCGTAGGGAGCAAACAGGATTAGATACCCTGGTAGTCTACGCTGTAAACGATGCACACTTGGTGTTAATCGAAAGGTTAGTACCGAAGCTAACGTGTTAAGTGTGCCGCCTGGGGAGTATGTTCGCAAGAATGAAACTCAAAGGAATTGACGGGGGCCCGCACAAGCGGTGGAGCATGTGGTTTAATTCGATGATACGCGAGGAACCTTACCAGGGCTTGACATATACAGGATATAGTTAGAGATAACTATTCCCCGTTTGGGGTCTGTATACAGGTGCTGCATGGTTGTCGTCAGCTCGTGCCGTGAGGTGTTGGGTTAAGTCCCGCAACGAGCGCAACCCTTGTTGTCTGTTACCAGCATGTAAAGATGGGGACTCAGACGAGACTGCCGGTGATAAGCCGGAGGAAGGTGAGGATGACGTCAAATCATCATGGCCCTTATGTCCTGGGCTACACACGTGCTACAATGGCCTGTACAAAGCGATGCGAAACAGTGATGTGAAGCAAAACGCAG-AAAGCAGGTCTCAGTCCAGATTGAAGTCTGAAACTCGGCTTCATGAAGTTGGAATCGCTAGTAATCGTATATCAGAATGATACGGTGAATACGTTCTCGGGCCTTGTACACACCGCCCGTCACACCACCC |
| *Borrelia* sp. | BJ164 | ON365960 | *rrs* | AGTCAAACGGAATGTAGCAATACATTCAGTGACGAACGGGTGAGTAACGCGTGGATAATCTACCTACGAGATGGGGATAACTATTAGAAATAGTAGCTAATACCGAATAAAGTCAATTGAGGTGTCAATTGATGAAAGGAAGCCTTTAAAGCTTCGCTTGTAGATGAGTCTGCGTCTTATTAGCTAGTTGGTAGGGTAAGAGCCTACCAAGGCtATGATAAGTAACCGGCCTGAGAGGGTGATCGGTCACACTGGAACTGAGATACGGTCCAGACTCCTACGGGAGGCAGCAGCTAAGAATCTTCCGCAATGGGCGAAAGCCTGACGGAGCGACACTGCGTGAACGAAGAAGGTCGAAAGATTGTAAAGTTCTTTTATAAATGAGGAATAAGCTTTGTAGGAAATGACAAAATGATGACGTTAGTTTATGAATAAGCCCCGGCTAATTACGTGCCAGCAGCCGCGGTAATACGTAAGGGGCGAGCGTTGTTCGGGATCATTGGGCGTAAAGGGTGAGTAGGCGGATATATAAGTCTATGTGTAAAATACCACAGCTCAACTGTGGAACTATGCTAGAAACTGCATGACTAGAGTCTGATAGGGGAAGTTAGAATTCCTGGTGTAAGGGTGGAATCTGTTGATATCAGGAAGAATACCAGAGGCGAAGGCGAACTTCTAGGTCAAGACTGACGCTGAGTCACGAAAGCGTAGGGAGCAAACAGGATTAGATACCCTGGTAGTCTACGCTGTAAACGATGCACACTGGGTGTTAATCGAAAGATTAGTACCGAAGCTAACGTGTTAAGTGTGCCGCCTGGGGAGTATGCTCGCAAGAGTGAAACTCAAAGGAATTGACGGGGGCCCGCACAAGCGGTGGAGCATGTGGTTTAATTCGATGATACGCGAGGAACCTTACCAGGGCTTGACATATACAGGATATAGTTAGAGATAACTACTCCCCGTTTGGGGTCTGTATACAGGTGCTGCATGGTTGTCGTCAGCTCGTGCTGTGAGGTGTTGGGTTAAGTCCCGCAACGAGCGCAACCCTTATTGTCTGTTACCAGCATGTAAAGATGGGGACTCAGACGAGACTGCCGGTGATAAGCCGGAGGAAGGTGAGGATGACGTCAAATCATCATGGCCCTTATGTCCTGGGCTACACACGTGCTACAATGGCCTGTACAAAGCGATGCGAAACAGTGATGTGAAGCAAAACGCAG-AAAGCAGGTCTCAGTCCAGATTGAAGTCTGAAACTCGACTTCATGAAGTTGGAATCGCTAGTAATCGTATATCAGAATGATACGGTGAATACGTTCTCGGGCCTTGTACACACCGCCCGTCACACCACCC |
| *Borrelia theileri* | HN1F2 | ON059603 | *rrs* | CGCTGTAAACGATGCACACTTGGTGTTAATCGAAAGGTTAGTACCGAAGCTAACGTGTTAAGTGTGCCGCCTGGGGAGTATGCTCGCAAGAGTGAAACTCAAAGGAATTGACGGGGGCCCGCACAAGCGGTGGAGCATGTGGTTTAATTCGATGATACGCGAGGAACCTTACCAGGGCTTGACATATACAGGATATAGTTAGAGATAACTACTCCCCTTTTGGGGTCTGTATACAGGTGCTGCATGGTTGTCGTCAGCTCGTGCTGTGAGGTGTTGGGTTAAGTCCCGCAACGAGCGCAACCCTTGTTGTCTGTTACCAGCATGTAAAGATGGGGACTCAGACGAGACTGCCG |
| *Borrelia miyamotoi* | HN3G11 | ON059604 | *rrs* | CGCTGTAAACGATGCACACTTGGTGTTAATCGAAAGGTTAGTACCGAAGCTAACGTGTTAAGTGTGCCGCCTGGGGAGTATGTTCGCAAGAATGAAACTCAAAGGAATTGACGGGGGCCCGCACAAGCGGTGGAGCATGTGGTTTAATTCGATGATACGCGAGGAACCTTACCAGGGCTTGACATATACAGGATATAGTTAGAGATAACTATTCCCCGTTTGGGGTCTGTATACAGGTGCTGCATGGTTGTCGTCAGCTCGTGCCGTGAGGTGTTGGGTTAAGTCCCGCAACGAGCGCAACCCTTGTTGTCTGTTACCAGCATGTAAAGATGGGGACTCAGACGAGACTGCCG |
| *Borrelia theileri* | GD4E5 | ON059605 | *rrs* | CGCTGTAAACGATGCACACTTGGTGTTAATCGAAAGGTTAGTACCGAAGCTAACGTGTTAAGTGTGCCGCCTGGGGAGTATGCTCGCAAGAGTGAAACTCAAAGGAATTGACGGGGGCCCGCACAATCGGTGGAGCATGTGGTTTAATTCGATGATACTCGAGGAACCTTACCAGGGCTTGACATATACAGGATATAGTTAGAGATAACTACTCCCCTTTTGGGGTCTGTATACAGGTGCTGCATGGTTGTCGTCAGCTCGTGCTGTGAGGTGTTGGGTTAAGTCCCGCAACGAGCGCAACCCTTGTTGTCTGTTACCAGCATGTAAAGATGGGGACTCAGACGAGACTGCCG |
| *Borrelia theileri* | GD4F9 | ON059606 | *rrs* | CGCTGTAAACGATGCACACTTGGTGTTAATCGAAAGGTTAGTACCGAAGCTAACGTGTTAAGTGTGCCGCCTGGGGAGTATGCTCGCAAGAGTGAAACTCAAAGGAATTGACGGGGGCCCGCACAAGCGGTGGAGCATGTGGTTTAATTCGATGATACGCGAGGAACCTTACCAGGGCTTGACATATACAGGATATAGTTAGAGATAACTACTCCCCTTTTGGGGTCTGTATACAGGTGCTGCATGGTTGTCGTCAGCTCGTGCTGTGAGGTGTTGGGTTAAGTCCCGCAACGAGCGCAACCCTTGTTGTCTGTTACCAGCATGTAAAGATGGGGACTCAGACGAGACTGCCG |
| *Borrelia* sp. | HLJ6A12 | ON059607 | *rrs* | CGCTGTAAACGATGCACACTTGGTGTTAATCGAAAGATTAGTACCGAAGCTAACGTGTTAAGTGTGCCGCCTGGGGAGTATGTTCGCAAGAGTGAAACTCAAAGGAATTGACGGGGGCCCGCACAAGCGGTGGAGCATGTGGTTTAATTCGATGATACGCGAGGAACCTTACCAGGGCTTGACATATACAGGATATAGTTAGAGATAACTATTCCCCGTTTGGGGTCTGTATACAGGTGCTGCATGGTTGTCGTCAGCTCGTGCTGTGAGGTGTTGGGTTAAGTCCCGCAACGAGCGCAACCCTTATTGTCTGTTACCAGCATGTAAAGATGGGGACTCAGACGAGACTGCCG |
| *Borrelia miyamotoi* | HLJ7G8 | ON059608 | *rrs* | CGCTGTAAACGATGCACACTTGGTGTTAATCGAAAGGTTAGTACCGAAGCTAACGTGTTAAGTGTGCCGCCTGGGGAGTATGTTCGCAAGAATGAAACTCAAAGGAATTGACGGGGGCCCGCACAAGCGGTGGAGCATGTGGTTTAATTCGATGATACGCGAGGAACCTTACCAGGGCTTGACATATACAGGATATAGTTAGAGATAACTATTCCCCGTTTGGGGTCTGTATACAGGTGCTGCATGGTTGTCGTCAGCTCGTGCCGTGAGGTGTTGGGTTAAGTCCCGCAACGAGCGCAACCCTTGTTGTCTGTTACCAGCATGTAAAGATGGGGACTCAAACAAGACTGCCG |
| *Borrelia miyamotoi* | HLJ7H10 | ON059609 | *rrs* | CGCTGTAAACGATGCACACTTGGTGTTAATCGAAAGATTAGTACCGAAGCTAACGTGTTAAGTGTGCCGCCTGGGGAGTATGCTCGCAAGAGTGAAACTCAAAGGAATTGACGGGGGCCCGCACAAGCGGTGGAGCATGTGGTTTAATTCGATGATACGCGAGGAACCTTACCAGGGCTTGACATATACAGGATATAGTTAGAGATAACTACTCCCCGTTTGGGGTCTGTATACAGGTGCTGCATGGTTGTCGTCAGCTCGTGCTGTGAGGTGTTGGGTTAAGTCCCGCAACGAGCGCAACCCTTATTGTCTGTTACCAGCATGTAAAGATGGGGACTCAGACGAGACTGCCG |
| *Borrelia miyamotoi* | JL8C8 | ON059610 | *rrs* | CGCTGTAAACGATGCACACTTGGTGTTAATCGAAAGGTTAGTACCGAAGCTAACGTGTTAAGTGTGCCGCCTGGGGAGTATGTTCGCAAGAATGAAACTCAAAGGAATTGACGGGGGCCCGCACAAGCGGTGGAGCATGTGGTTTAATTCGATGATACGCGAGGAACCTTACCAGGGCTTGACATATACAGGATATAGTTAGAGATAACTATTCCCCGTTTGGGGTCTGTATACAGGTGCTGCATGGTTGTCGTCAGCTCGTGCCGTGAGGTGTTGGGTTAAGTCCCGCAACGAGCGCAACCCTTGTTGTCTGTTACCAGCATGTAAAGATGGGGACTCAGACGAGACTGCCG |
| *Borrelia miyamotoi* | JL8G8 | ON059611 | *rrs* | CGCTGTAAACGATGCACACTTGGTGTTAATCGAAAGGTTAGTACCGAAGCTAACGTGTTAAGTGTGCCGCCTGGGGAGTATGTTCGCAAGAATGAAACTCAAAGGAATTGACGGGGGCCCGCACAAGCGGTGGAGCATGTGGTTTAATTCGATGATACGCGAGGAACCTTACCAGGGCTTGACATATACAGGATATAGTTAGAGATAACTATTCCCCGTTTGGGGTCTGTATACAGGTGCTGCATGGTTGTCGTCAGCTCGTGCCGTGAGGTGTTGGGTTAAGTCCCGCAACGAGCGCAACCCTTGTTGTCTGTTACCAGCATGTAAAGATGGGGACTCAAACAAGACTGCCG |
| *Borrelia miyamotoi* | JL8D12 | ON059612 | *rrs* | CGCTGTAAACGATGCACACTTGGTGTTAATCGAAAGGTTAGTACCGAAGCTAACGTGTTAAGTGTGCCGCCTGGGGAGTATGTTCGCAAGAATGAAACTCAAAGGAATTGACGGGGGCCCGCACAAGCGGTGGAGCATGTGGTTTAATTCGATGATACGCGAGGAACCTTACCAGGGCTTGACATATACAGGATATAGTTAGAGATAACTATTCCCCGTTTGGGGTCTGTATACAGGTGCTGCATGGTTGTCGTCAGCTCGTGCCGTGAGGTGTTGGGTTAAGTCCCGCAACGAGCGCAACCCTTGTTGTCTGTTACCAGCATGTAAAGATGGGGACTCAGACAAGACTGCCG |
| *Borrelia miyamotoi* | JL8G11 | ON059613 | *rrs* | CGCTGTAAACGATGCACACTTGGTGTTAATCGAAAGGTTAGTACCGAAGCTAACGTGTTAAGTGTGCCGCCTGGGGAGTATGTTCGCAAGAATGAAACTCAAAGGAATTGACGGGGGCCCGCACAAGCGGTGGAGCATGTGGTTTAATTCGATGATACGCGAGGAACCTTACCAGGGCTTGACATATACAGGATATAGTTAGAGATAACTATTCCCCGTTTGGGGTCTGTATACAGGTGCTGCATGGTTGTCGTCAGCTCGTGCTGTGAGGTGTTGGGTTAAGTCCCGCAACGAGCGCAACCCTTGTTGTCTGTTACCAGCATGTAAAGATGGGGACTCAGACGAGACTGCCG |
| *Borrelia miyamotoi* | JL9H1 | ON059614 | *rrs* | CGCTGTAAACGATGCACACTTGGTGTTAATCGAAAGGTTAGTACCGAAGCTAACGTGTTAAGTGTGCCGCCTGGGGAGTATGTTCGCAAGAATGAAACTCAAAGGAATTGACGGGGGCCCGCACAAGCGGTGGAGCATGTGGTTTAATTCGATGATACGCGAGGAACCTTACCAGGGCTTGACATATACAGGATATAGTTAGAGATAACTATTCCCCGTTTGGGGTCTGTATACAGGTGCTGCATGGTTGTCGTCAGCTCGTGCCGTGAGGTGTTGGGTTAAGTCCCGCAACGAGCGCAACCCTTGTTGTCTGTTACCAGCATGTAAAGATGGGGACTCAGACGAGACTGCCG |
| *Borrelia* sp. | HLJ9H6 | ON059615 | *rrs* | CGCTGTAAACGATGCACACTTGGTGTTAATCGAAAGATTAGTACCGAAGCTAACGTGTTAAGTGTGCCGCCTGGGGAGTATGCTCGCAAGAGTGAAACTCAAAGGAATTGACGGGGGCCCGCACAAGCGGTGGAGCATGTGGTTTAATTCGATGATACGCGAGGAACCTTACCAGGGCTTGACATATACAGGATATAGTTAGAGATAACTACTCCCCGTTTGGGGTCTGTATACAGGTGCTGCATGGTTGTCGTCAGCTCGTGCTGTGAGGTGTTGGGTTAAGTCCCGCAACGAGCGCAACCCTTATTGTCTGTTACCAGCATGTAAAGATGGGGACTCAGACGAGACTGCCG |
| *Borrelia miyamotoi* | HLJ11F5 | ON059616 | *rrs* | CGCTGTAAACGATGCACACTTGGTGTTAATCGAAAGGTTAGTACCGAAGCTAACGTGTTAAGTGTGCCGCCTGGGGAGTATGTTCGCAAGAATGAAACTCAAAGGAATTGACGGGGGCCCGCACAAGCGGTGGAGCATGTGGTTTAATTCGATGATACGCGAGGAACCTTACCAGGGCTTGACATATACAGGATATAGTTAGAGATAACTATTCCCCGTTTGGGGTCTGTATACAGGTGCTGCATAGTTGTCGTCAGCTCGTGCCGTGAGGTGTTGGGTTAAGTCCCGCAACGAGCGCAACCCTTGTTGTCTGTTACCAGCATGTAAAGATGGGGACTCAGACGAGACTGCCG |
| *Borrelia* sp. | HLJ11C8 | ON059617 | *rrs* | CGCTGTAAACGATGCACACTTGGTGTTAATCGAAAGGTTAGTACCGAAGCTAACGTGTTAAGTGTGCCGCCTGGGGAGTATGCTCGCAAGAGTGAAACTCAAAGGAATTGACGGGGGCCCGCACAAGCGGTGGAGCATGTGGTTTAATTCGATGATACGCGAGGAACCTTACCAGGGCTTGACATATACAGGATATAGTTAGAGATAACTACTCCCCGTTTGGGGTCTGTATACAGGTGCTGCATGGTTGTCGTCAGCTCGTGCTGTGAGGTGTTGGGTTAAGTCCCGCAACGAGCGCAACCCTTATTGTCTGTTACCAGCATGTAAAGATGGGGACTCAGACGAGACTGCCG |
| *Borrelia miyamotoi* | LN15E1 | ON059618 | *rrs* | CGCTGTAAACGATGCACACTTGGTGTTAATCGAAAGGTTAGTACCGAAGCTAACGTGTTAAGTGTGCCGCCTGGGGAGTATGTTCGCCGGAATGAAACTCAAAGGAATTGACGGGGGCCCGCACAAGCGGTGGAGCATGTGGTTTAATTCGATGATACGCGAGGAACCTTACCAGGGCTTGACATATACAGGATATAGTTAGAGATAACTATTCCCCGTTTGGGGTCTGTATACAGGTGCTGCATGGTTGTCGTCAGCTCGTGCCGTGAGGTGTTGGGTTAAGTCCCGCAACGAGCGCAACCCTTGTTGTCTGTTACCAGCATGTAAAGATGGGGACTCAGACGAGACTGCCG |
| *Borrelia miyamotoi* | HLJ16G2 | ON059619 | *rrs* | CGCTGTAAACGATGCACACTTGGTGTTAATCGAAAGGTTAGTACCGAAGCTAACGTGTTAAGTGTGCCGCCTGGGGAGTATGTTCGCAAGAATGAAACTCAAAGGAATTGACGGGGGCCCGCACAAGCGGTGGAGCATGTGGTTTAATTCGATGATACGCGAGGAACCTTACCAGGGCTTGACATATACAGGATATAGTTAGAGATAACTATTCCCCGTTTGGGGTCTGTATACAGGTGCTGCATGGTTGTCGTCAGCTCGTGCCGTGAGGTGTTGGGTTAAGTCCCGCAACGAGCGCAACCCTTGTTGTCTGTTACCAGCATGTAAAGATGGGGACTCAGACGAGACTGCCG |
| *Borrelia miyamotoi* | LN16F7 | ON059620 | *rrs* | CGCTGTAAACGATGCACACTTGGTGTTAATCGAAAGGTTAGTACCGAAGCTAACGTGTTAAGTGTGCCGCCTGGGGAGTATGTTCGCAAGAATGAAACTCAAAGGAATTGACGGGGGCCCGCACAAGCGGTGGAGCATGTGGTTTAATTCGATGATACGCGAGGAACCTTACCAGGGCTTGACATATACAGGATATAGTTAGAGATAACTATTCCCCGTTTGGGGTCTGTATACAGGTGCTGCATGGTTGTCGTCAGCTCGTGCCGTGAGGTGTTGGGTTAAGTCCCGCAACGAGCGCAACCCTTGTTGTCTGTTACCAGCATGTAAAGATGGGGACTCAGACGAGACTGCCG |
| *Borrelia* sp. | NMG9D11 | ON059621 | *rrs* | CGCTGTAACCGATGCACACTTGGTGTTAATCGAAAGATTAGTACCGAAGCTAACGTGTTAAGTGTGCCGCCTGGGGAGTATGCTCGCAAGAGTGAAACTCAAAGGAATTGACGGGGGCCCGCACAAGCGGTGGAGCATGTGGTTTAATTCGATGATACGCGAGGAACCTTACCAGGGCTTGACATATACAGGATATAGTTAGAGATAACTACTCCCCGTTTGGGGTCTGTATACAGGTGCTGCATGGTTGTCGTCAGCTCGTGCTGTGAGGTGTTGGGTTAAGTCCCGCAACGAGCGCAACCCTTATTGTCTGTTACCAGCATGTAAAGATGGGGACTCAGACGAGACTGCCG |
| *Borrelia* sp. | NMG10G3 | ON059622 | *rrs* | CGCTGTAAACGATGCACACTTGGTGTTAATCGAAAGATTAGTACCGAAGCTAACGTGTTAAGTGTGCCGCCTGGGGAGTATGCTCGCAAGAGTGAAACTCAAAGGAATTGACGGGGGCCCGCACAAGCGGTGGAGCATGTGGTTTAATTCGATGATACGCGAGGAACCTTACCAGGGCTTGACATATACAGGATATAGTTAGAGATAACTACTCCCCGTTTGGGGTCTGTATACAGGTGCTGCATGGTTGTCGTCAGCTCGTGCTGTGAGGTGTTGGGTTAAGTCCCGCAACGAGCGCAACCCTTATTGTCTGTTACCAGCATGTAAAGATGGGGACTCAGACGAGACTGCCG |
| *Borrelia* sp. | NMG10H9 | ON059623 | *rrs* | CGCTGTAAACGATGCACACTTGGTGTTAATCGAAAGATTAGTACCGAAGCTAACGTGTTAAGTGTGCCGCCTGGGGAGTATGCTCGCAAGAGTGAAACTCAAAGGAATTGACGGGGGCCCGCACAAGCGGTGGAGCATGTGGTTTAATTCGATGATACGCGAGGAACCTTACCAGGGCTTGACATATACAGGATATAGTTAGAGATAACTACTCCCCGTTTGGGGTCTGTATACAGGTGCTGCATGGTTGTCGTCAGCTCGTGCTGTGAGGTGTTGGGTTAAGTCCCGCAACGAGCGCAACCCTTATTGTCTGTTACCAGCATGTAAAGATGGGGACTCAGACGAGACTGCCG |
| *Borrelia miyamotoi* | NMG10H11 | ON059624 | *rrs* | CGCTGTAAACGATGCACACTTGGTGTTAATCGAAAGGTTAGTACCGAAGCTAACGTGTTAAGTGTGCCGCCTGGGGAGTATGTTCGCAAGAATGAAACTCAAAGGAATTGACGGGGGCCCGCACAAGCGGTGGAGCATGTGGTTTAATTCGATGATACGCGAGGAACCTTACCAGGGCTTGACATATACAGGATATAGTTAGAGATAACTATTCCCCGTTTGGGGTCTGTATACAGGTGCTGCATGGTTGTCGTCAGCTCGTGCCGTGAGGTGTTGGGTTAAGTCCCGCAACGAGCGCAACCCTTGTTGTCTGTTACCAGCATGTAAAGATGGGGACTCAGACGAGACTGCCG |
| *Borrelia* sp. | HNQW5 | ON059625 | *rrs* | CGCTGTAAACGATGCACACTTGGTGTTAATCGAAAGGTTAGTACCGAAGCTAACGTGTTAAGTGTGCCGCCTGGGGAGTATGTTCGCAAGAATGAAACTCAAAGGGATTGACGGGGGCCCGCACAAGCGGTGGAGCATGTGGTTTAATTCGATGATACGCGAGGAACCTTACCAGGGCTTGACATATACAGGATATAGTTAGAGATAACTATTCCCCGTTTGGGGTCTGTATACAGGTGCTGCATGGTTGTCGTCAGCTCGTGCCGTGAGGTGTTGGGTTAAGTCCCGCAACGAGCGCAACCCTTGTTGTCTGTTACCAGCATGTAAAGATGGGGACTCAGACGAGACTGCCG |
| *Borrelia miyamotoi* | HNPT012 | ON059626 | *rrs* | CGCTGTAAACGATGCACACTTGGTGTTAATCGAAAGGTTAGTACCGAAGCTAACGTGTTAAGTGTGCCGCCTGGGGAGTATGTTCGCAAGAATGAAACTCAAAGGAATTGACGGGGGCCCGCACAAGCGGTGGAGCATGTGGTTTAATTCGATGATACGCGAGGAACCTTACCAGGGCTTGACATATACAGGATATAGTTAGAGATAACTATTCCCCGTTTGGGGTCTGTATACAGGTGCTGCATGGTTGTCGTCAGCTCGTGCCGTGAGGTGTTGGGTTAAGTCCCGCAACGAGCGCAACCCTTGTTGTCTGTTACCAGCATGTAAAGATGGGGACTCAGACGAGACTGCCG |
| *Borrelia miyamotoi* | SDY9 | ON059627 | *rrs* | CGCTGTAAACGATGCACACTTGGTGTTAATCGAAAGGTTAGTACCGAAGCTAACGTGTTAAGTGTGCCGCCTGGGGAGTATGTTCGCAGGAATGAAACTCAAAGGAATTGACGGGGGCCCGCACAAGCGGTGGAGCATGTGGTTTAATTCGATGATACGCGAGGAACCTTACCAGGGCTTGACATATACAGGATATAGTTAGAGATAACTATTCCCCGTTTGGGGTCTGTATACAGGTGCTGCATGGTTGTCGTCAGCTCGTGCCGTGAGGTGTTGGGTTAAGTCCCGCAACGAGCGCAACCCTTGTTGTCTGTTACCAGCATGTAAAGATGGGGACTCAGACGAGACTGCCG |
| *Borrelia miyamotoi* | SDBY23 | ON059628 | *rrs* | CGCTGTAAACGATGCACACTTGGTGTTAATCGAAAGATTAGTACCGAAGCTAACGTGTTAAGTGTGCCGCCTGGGGAGTATGTTCGCAAGAATGAAACTCAAAGGAATTGACGGGGGCCCGCACAAGCGGTGGAGCATGTGGTTTAATTCGATGATACGCGAGGAACCTTACCAGGGCTTGACATATACAGGATATAGTTAGAGATAACTATTCCCCGTTTGGGGTCTGTATACAGGTGCTGCATGGTTGTCGTCAGCTCGTGCCGTGAGGTGTTGGGTTAAGTCCCGCAACGAGCGCAACCCTTGTTGTCTGTTACCAGCATGTAAAGATGGGGACTCAGACGAGACTGCCG |
| *Borrelia miyamotoi* | SDWY24 | ON059629 | *rrs* | CGCTGTAAACGATGCACACTTGGTGTTAATCGAAAGGTTAGTACCGAAGCTAACGTGTTAAGTGTGCCGCCTGGGGAGTATGTTCGCAAGAATGAAACTCAAAGGAATTGACGGGGGCCCGCACAAGCAGTGGAGCATGTGATTTAATTCGATGATACGCGAGGAACCTTACCAGGGCTTGACATATACAGGATATAGTTAGAGATAACTATTCCCCGTTTGGGGTCTGTATACAGGTGCTGCATGGTTGTCGTCAGCTCGTGCCGTGAGGTGTTGGGTTAAGTCCCGCAACGAGCGCAACCCTTGTTGTCTGTTACCAGCATGTAAAGATGGGGACTCAGACGAGACTGCCG |
| *Borrelia* sp. | NMGBF43 | ON059630 | *rrs* | CGCTGTAAACGATGCACACTTGGTGTTAATCGAAAGATTAGTACCGAAGCTAACGTGTTAAGTGTGCCGCCTGGGGAGTATGCTCGCAAGAGTGAAACTCAAAGGAATTGACGGGGGCCCGCACAAGCGGTGGAGCATGTGGTTTAATTCGATGATACGCGAGGAACCTTACCAGGGCTTGACATATACAGGATATAGTTAGAGATAACTACTCCCCGTTTGGGGTCTGTATACAGGTGCTGCATGGTTGTCGTCAGCTCGTGCTGTGAGGTGTTGGGTTAAGTCCCGCAACGAGCGCAACCCTTATTGTCTGTTACCAGCATGTAAAGATGGGGACTCAGACGAGACTGCCG |
| *Borrelia miyamotoi* | NMGBF77 | ON059631 | *rrs* | CGCTGTAAACGATGCACACTTGGTGTTAATCGAAAGGTTAGTACCGAAGCTAACGTGTTAAGTGTGCCGCCTGGGGAGTATGTTCGCGAGAATGAAACTCAAAGGAATTGACGGGGGCCCGCACAAGCGGTGGAGCATGTGGTTTAATTCGATGATACGCGAGGAACCTTACCAGGGCTTGACATATACAGGATATAGTTAGAGATAACTATTCCCCGTTTGGGGTCTGTATACAGGTGCTGCATGGTTGTCGTCAGCTCGTGCCGTGAGGTGTTGGGTTAAGTCCCGCAACGAGCGCAACCCTTGTTGTCTGTTACCAGCATGTAAAGATGGGGACTCAGACGAGACTGCCG |
| *Borrelia miyamotoi* | NMGBF95 | ON059632 | *rrs* | CGCTGTAAACGATGCACACTTGGTGTTAATCGAAAGGTTAGTACCGAAGCTAACGTGTTAAGTGTGCCGCCTGGGGAGTATGTTCGCAAGAATGAAACTCAAAGGAATTGACGGGGGCCCGCACAAGCGGTGGAGCATGTGGTTTAATTCGATGATACGCGAGGAACCTTACCAGGGCTTGACATATACAGGATATAGTTAGAGATAACTATTCCCCGTTTGGGGTCTGTATACAGGTGCTGCATGGTTGTCGTCAGCTCGTGCCGTGAGGTGTTGGGTTAAGTCCCGCAACGAGCGCAACCCTTGTTGTCTGTTACCAGCATGCAAAGATGGGGACTCAGACGAGACTGCCG |
| *Borrelia miyamotoi* | NMGBM21 | ON059633 | *rrs* | CGCTGTAAACGATGCACACTTGGTGTTAATCGAAAGGTTAGTACCGAAGCTAACGTGTTAAGTGTGCCGCCTGGGGAGTATGTTCGCAAGAATGAAACTCAAAGGAATTGACGGGGGCCCGCACAAGCGGTGGAGCATGTGGTTTAATTCGATGATACGCGAGGAACCTTACCAGGGCTTGACATATACAGGATATAGTTAGAGATAACTATTCCCCGTTTGGGGTCTGTATACAGGTGCTGCATGGTTGTCGTCAGCTCGTGCCGTGAGGTGTTGGGTTAAGTCCCGCAACGAGCGCAACCCTTGTTGTCTGTTACCAGCATGTAAAGATGGGGACTCAGACGAGACTGCCG |
| *Borrelia* sp. | SDJN171 | ON059634 | *rrs* | CGCTGTAAACGATGCACACTTGGTGTTAATCGAAAGATTAGTACCGAAGCTAACGTGTTAAGTGTGCCGCCTGGGGAGTATGCTCGCAAGAGTGAAACTCAAAGGAATTGACGGGGGCCCGCACAAGCGGTGGAGCATGTGGTTTAATTCGATGATACGCGAGGAACCTTACCAGGGCTTGACATATACAGGATATAGTTAGAGATAACTACTCCCCGTTTGGGGTCTGTATACAGGTGCTGCATGGTTGTCGTCAGCTCGTGCTGTGAGGTGTTGGGTTAAGTCCCGCAACGAGCGCAACCCTTGTTGTCTGTTACCAGCATGTAAAGATGGGGACTCAGACGAGACTGCCG |
| *Borrelia miyamotoi* | SD32H10 | ON059635 | *rrs* | CGCTGTAAACGATGCACACTTGGTGTTAATCGAAAGATTAGTACCGAAGCTAACGTGTTAAGTGTGCCGCCTGGGGAGTATGTTCGCAAGAGTGAAACTCAAAGGAATTGACGGGGGCCCGCACAAGCGGTGGAGCATGTGGTTTAATTCGATGATACGCGAGGAACCTTACCAGGGCTTGACATATACAGGATATAGTTAGAGATAACTATTCCCCGTTTGGGGTCTGTATACAGGTGCTGCATGGTTGTCGTCAGCTCGTGCCGTGAGGTGTTGGGTTAAGTCCCGCAACGAGCGCAACCCTTGTTGTCTGTTACCAGCATGTAAAGATGGGGACTCAGACGAGACTGCCG |
| *Borrelia miyamotoi* | SD33H1 | ON059636 | *rrs* | CGCTGTAAACGATGCACACTTGGTGTTAATCGAAAGGTTAGTACCGAAGCTAACGTGTTAAGTGTGCCGCCTGGGGAGTATGTTCGCAAGAATGAAACTCAAAGGAATTGACGGGGGCCCGCACAAGCGGTGGAGCATGTGGTTTAATTCGATGATACGCGAGGAACCTTACCAGGGCTTGACATATACAGGATATAGTTAGAGATAACTATTCCCCGTTTGGGGTCTGTATACAGGTGCTGCATGGTTGTCGTCAGCTCGTGCCGTGAGGTGTTGGGTTAAGTCCCGCAACGAGCGCAACCCTTGTTGTCTGTTACCAGCATGTAAAGATGGGGACTCAGACGAGACTGCCG |
| *Borrelia* sp. | NMG105 | ON059637 | *rrs* | CGCTGTAAACGATGCACACTTGGTGTTAATCGAGAGATTAGTACCGAAGCTAACGTGTTAAGTGTGCCGCCTGGGGAGTATGCTCGCAAGAGTGAAACTCAAAGGAATTGACGGGGGCCCGCACAAGCGGTGGAGCATGTGGTTTAATTCGATGATACGCGAGAAACCTTACCAGGGCTTGACATATACAGGATGTAGTTAGAGATAATTATTCCCCGTTTGGGGTCTGTATACAGGTGCTGCATGGTTGTCGTCAGCTCGTGCTGTGAGGTGTTGGGTTAAGTCCCGCAACGAGCGCAACCCTTATTGTCTGTTACCAGCATGTAAAGATGGGGACTCAGACGAGACTGCCG |
| *Borrelia miyamotoi* | SD4195 | ON059638 | *rrs* | CGCTGTAAACGATGCACACTTGGTGTTAATCGAAAGGTTAGTACCGAAGCTAACGTGTTAAGTGTGCCGCCTGGGGAGTATGTTCGCAAGAATGAAACTCAAAGGAATTGACGGGGGCCCGCACAAGCGGTGGAGCATGTGGTTTAATTCGATGATACGCGAGGAACCTTACCAGGGCTTGACATATACAGGATATAGTTAGAGATAACTATTCCCCGTTTGGGGTCTGTATACAGGTGCTGCATGGTTGTCGTCAGCTCGTGCCGTGAGGTGTTGGGTTAAGTCCCGCAACGAGCGCAACCCTTGTTGTCTGTTACCAGCATGTAAAGATGGGGACTCAGACGAGACTGCCG |
| *Borrelia* sp. | HN21413 | ON059639 | *rrs* | CGCTGTAAACGATGCACACTTGGTGTTAATCGAAAGATTAGTACCGAAGCTAACGTGTTAAGTGTGCCGCCTGGGGAGTATGCTCGCAAGAGTGAAACTCAAAGGAATTGACGGGGGCCCGCACAAGCGGTGGAGCATGTGGTTTAATTCGATGATACGCGAGGAACCTTACCAGGGCTTGACATATACAGGATATAGTTAGAGATAACTACTCCCCGTTTGGGGTCTGTATACAGGTGCTGCATGGTTGTCGTCAGCTCGTGCTGTGAGGTGTTGGGTTAAGTCCCGCAACGAGCGCAACCCTTATTGTCTGTTACCAGCATGTAAAGATGGGGACTCAGACGAGACTGCCG |
| *Borrelia* sp. | HN21210 | ON059640 | *rrs* | CGCTGTAAACGATGCACACTTGGTGTTAATCGAAAGATTAGTACCGAAGCTAACGTGTTAAGTGTGCCGCCTGGGGAGTATGCTCGCAAGAGTGAAACTCAAAGGAATTGACGGGGGCCCGCACAAGCGGTGGAGCATGTGGTTTAATTCGATGATACGCGAGAAACCTTACCAGGGCTTGACATATACAGGATGTAGTTAGAGATAATTATTCCCCGTTTGGGGTCTGTATACAGGTGCTGCATGGTTGTCGTCAGCTCGTGCTGTGAGGTGTTGGGTTAAGTCCCGCAACGAGCGCAACCCTTATTGTCTGTTACCAGCATGTAAAGATGGGGACTCAGACGAGACTGCCG |
| *Borrelia theileri* | YN1B2 | ON059641 | *rrs* | CGCTGTAAACGATGCACACTTGGTGTTAATCGAAAGGTTAGTACCGAAGCTAACGTGTTAAGTGTGCCGCCTGGGGAGTATGCTCGCAAGAGTGAAACTCAAAGGAATTGACGGGGGCCCGCACAAGCGGTGGAGCATGTGGTTTAATTCGATGATACGCGAGGAACCTTACCAGGGCTTGACATATACAGGATATAGTTAGAGATAACTACTCCCCTTTTGGGGTCTGTATACAGGTGCTGCATGGTTGTCGTCAGCTCGTGCTGTGAGGTGTTGGGTTAAGTCCCGCAACGAGCGCAACCCTTGTTGTCTGTTACCAGCATGTAAAGATGGGGACTCAGACGAGACTGCCG |
| *Borrelia* sp. | BJ145 | ON059642 | *rrs* | CGCTGTAAACGATGCACACTTGGTGTTAATCGAAAGATTAGTACCGAAGCTAACGTGTTAAGTGTGCCGCCTGGGGAGTATGCTCGCAAGAGTGAAACTCAAAGGAATTGACGGGGGCCCGCACAAGCGGTGGAGCATGTGGTTTAATTCGATGATACGCGAGAAACCTTACCAGGGCTTGACATATACAGGATGTAGTTAGAGATAATTATTCCCCGTTTGGGGTCTGTATACAGGTGCTGCATGGTTGTCGTCAGCTCGTGCTGTGAGGTGTTGGGTTAAGTCCCGCAACGAGCGCAACCCTTATTGTCTGTTACCAGCATGTAAAGATGGGGACTCAGACGAGACTGCCG |
| *Borrelia* sp. | HN2148 | ON059643 | *rrs* | CGCTGTAAACGATGCACACTTGGTGTTAATCGAAAGATTAGTACCGAAGCTAACGTGTTAAGTGTGCCGCCTGGGGAGTATGCTCGCAAGAGTGAAACTCAAAGGAATTGACGGGGGCCCGCACAAGCGGTGGAGCATGTGGTTTAATTCGATGATACGCGAGGAACCTTACCAGGGCTTGACATATACAGGATATAGTTAGAGATAACTACTCCCCGTTTGGGGTCTGTATACAGGTGCTGCATGGTTGTCGTCAGCTCGTGCTGTGAGGTGTTGGGTTAAGTCCCGCAACGAGCGCAACCCTTATTGTCTGTTACCAGCATGTAAAGATGGGGACTCAGACGAGACTGCCG |
| *Borrelia miyamotoi* | HLJ6F5 | ON060662 | *flaB* | ATCTGATGATGCTGCTGGTATGGGTGTTGCTGGTAAGCTTAATTCACAAATTAGAGGATTGTCTCAAGCTTCTAGAAATACCTCAAAGGCTATAAATTTTATTCAAACAACAGAAGGAAATTTGAACGAGGTAGAGAAAGTATTAGTAAGAATGAAAGAACTTGCTGTTCAGTCTGGTAATGGTACATACTCAGATTCAGATAGAGGGTCTATTCAGATTGAAATTGAACAACTTACAGATGAAATAAACAGAATTGCTGATCAGGCTCAATACAACCAAATGCATATGTTATCTAATAAGTCAGCTGCTCAAAATGTAAAAACTGCTGAAGAGCTTGGAATGCAACCTGCAAAAATTAACACACCAGCATCATTGGCTGGATCACAAGCTTCATGGACATTGAGAGTGCATGTAGGTGCAAATCAGGATGAAGCAATTGCTGTCAATATTTATGCAGCTAATGTTGCAAATCTTTTTAATGGAGAAGGTGCTCAAGCAGCTCCAG |
| *Borrelia* sp. | HLJ9E4 | ON060663 | *flaB* | TTCTGATGATGCTGCTGGTATGGGTGTTGCTGGTAAGCTTAATTCTCAAATTAGGGGATTATCTCAAGCTTCTAGAAATACTTCAAAGGCTATAAATTTTATTCAAACAACAGAAGGAAATTTGAATGAAGTAGAGAAGATATTAGTTAGAATGAAAGAACTTGCTGTTCAGTCTGGTAATGGTACATACTCAGATGCAGACAGAGGTTCTATTCAAATTGAAATTGAACAACTTACAGATGAAATTAACAGAGTGGCTGATCAGGCTCAATATAACCAGATGCATATGTTATCTAACAAGTCATCTGCTCAAAATGTAAAAACTGCTGAAGAGCTTGGAATGCAACCTGCAAAAATTAACACACCAGCATCACTAACTGGAGCACAAGCTTCATGGACATTAAGAGTTCAAGTGGGTGCAAATCAGGATGAAGCAATTGCAGTTAATATTTTTTCAACTAATGTTGCAAATCTTTTTGGTGGAGAAGGTGCTCAAGCAGCTCCAG |
| *Borrelia* sp. | NMG10D10 | ON060664 | *flaB* | ATCTGATGATGCTGCTGGTATGGGTGTTGCTGGTAAGCTTAATTCTCAAATTAGGGGATTATCTCAAGCTTCTAGAAATACTTCAAAGGCTATAAATTTTATTCAAACAACAGAAGGAAATTTGAATGAAGTAGAGAAGATATTAGTTAGAATGAAAGAACTTGCTGTTCAGTCTGGTAATGGTACATACTCAGATGCAGACAGAGGTTCTATTCAAATTGAAATTGAACAACTTACAGATGAAATTAACAGAGTGGCTGATCAGGCTCAATATAACCAGATGCATATGTTATCTAACAAATCATCTGCTCAAAATGTAAAAACTGCTGAAGAGCTTGGAATGCAACCTGCAAAAATTAACACACCAGCATCACTAACTGGAGCACAAGCTTCATGGACATTAAGAGTTCAAGTGGGTGCAAATCAGGATGAAGCAATTGCAGTTAATATTTTTGCAACTAATGTTGCAAATCTTTTTGGTGGAGAAGGTGCTCAAGCAGCTCCAG |
| *Borrelia theileri* | GD4C9 | ON060665 | *flaB* | TCTGATGATGCTGCTGGTATGGGTGTTGCTGGTAAGCTTAATTCTCAAATTAGGGGATTATCTCAAGCTTCTAGAAATACTTCAAAGGCTATAAATTTTATTCAAACAACAGAAGGAAATTTGAATGAAGTAGAGAAGATATTAGTTAGAATGAAAGAACTTGCTGTTCAGTCTGGTAATGGTACATACTCAGATGCAGACAGAGGTTCTATTCAAATTGAAATTGAACAACTTACAGATGAAATTAACAGAGTGGCTGATCAGGCTCAATATAACCAGATGCATATGTTATCTAACAAATCATCTGCTCAAAATGTAAAAACTGCTGAAGAGCTTGGAATGCAACCTGCAAAAATTAACACACCAGCATCACTAACTGGAGCACAAGCTTCATGGACATTAAGAGTTCAAGTGGGTGCAAATCAGGATGAAGCAATTGCAGTTAATATTTTTGCAACTAATGTTGCAAATCTTTTTGGTGGAGAAGGTGTTCAAGCAGCTCCAGCTCAAGAGGGTGCACAACAGGAAGGAGTTCAACCAGCTCCAGCTCAAGGTGGAGTTAGCTCTCCTATTAATGTTACAACTGCTATTGATGCTAATACATCACTTACAAAGATTGAAGATGCTATTAGAATGATAACTGATCAAAGAGC |
| *Borrelia* sp. | NMG10E7 | ON060666 | *flaB* | TCTGATGATGCTGCTGGTATGGGTGTTGCTGGTAAGCTTAATTCTCAAATTAGGGGATTATCTCAAGCTTCTAGAAATACTTCAAAGGCTATAAATTTTATTCAAACAACAGAAGGAAATTTGAATGAAGTAGAGAAGATATTAGTTAGAATGAAAGAACTTGCTGTTCAGTCTGGTAATGGTACATACTCAGATGCAGACAGAGGTTCTATTCAAATTGAAATTGAACAACTTACAGATGAAATTAACAGAGTGGCTGATCAGGCTCAATATAACCAGATGCATATGTTATCTAACAAATCATCTGCTCAAAATGTAAAAACTGCTGAAGAGCTTGGAATGCAACCTGCAAAAATTAACACACCAGCATCACTAACTGGAGCACAAGCTTCATGGACATTAAGAGTTCAAGTGGGTGCAAATCAGGATGAAGCAATTGCAGTTAATATTTTTGCAACTAATGTTGCAAATCTTTTTGGTGGAGAAGGTGTTCAAGCAGCTCCAGCTCAAGAGGGTGCACAACAGGAAGGAGTTCAACCAGCTCCAGCTCAAGGTGGAGTTAGCTCTCCTATTAATGTTACAACTGCTATTGATGCTAATACATCACTTACAAAGATTGAAGATGCTATTAGAATGATAACTGATCAAAGAGC |
| *Borrelia* sp. | HLJ9C11 | ON060667 | *flaB* | TCTGATGATGCTGCTGGTATGGGTGTTGCTGGTAAGCTTAATTCTCAAATTAGGGGATTATCTCAAGCTTCTAGAAATACTTCAAAGGCTATAAATTTTATTCAAACAACAGAAGGAAATTTGAATGAAGTAGAGAAGATATTAGTTAGAATGAAAGAACTTGCTGTTCAGTCTGGTAATGGTACATACTCAGATGCAGACAGAGGTTCTATTCAAATTGAAATTGAACAACTTACAGATGAAATTAACAGAGTGGCTGATCAGGCTCAATATAACCAGATGCATATGTTATCTAACAAGTCATCTGCTCAAAATGTAAAAACTGCTGAAGAGCTTGGAATGCAACCTGCAAAAATTAACACACCAGCATCACTAACTGGAGCACAAGCTTCATGGACATTAAGAGTTCAAGTGGGTGCAAATCAGGATGAAGCAATTGCAGTTAATATTTTTTCAACTAATGTTGCAAATCTTTTTGGTGGAGAAGGTGTTCAAGCAGCTCCAGCTCAAGAGGGTGCACAACAGGAAGGAGTTCAACCAGCTCCAGCTCAAGGTGGAGTTAGCTCTCCTATTAATGTTACAACTGCTATTGATGCTAATACATCACTTACAAAGATTGAAGATGCTATTAGAATGATAACTGATCAAAGAGC |
| *Borrelia theileri* | HN1F2 | ON060668 | *flaB* | TCTGATGATGCTGCTGGTATGGGTGTTGCTGGTAAGCTTAATTCTCAAATTAGGGGATTATCTCAAGCTTCTAGAAATACTTCAAAGGCTATAAATTTTATTCAAACAACAGAAGGAAATTTGAATGAGGTGGAGAAGATATTAGTTAGAATGAAAGAACTTGCTGTTCAATCTGGTAATGGTACATACTCAGATGCAGACAGAGGTTCTATTCAAATTGAAATTGAACAACTTACAGATGAAATTAACAGAGTTGCTGATCAGGCTCAATATAACCAGATGCACATGTTATCTAACAAGTCATCTGCTCAAAATGTAAGAACTGCTGAAGAGCTTGGAATGCAACCTGCAAAAATTAATACACCAGCATCACTAACTGGATCACAAGCTTCATGGACATTGAGAGTTCAAGTAGGTGCAAATCAAGATGAAGCAATTGCTGTTAATATTTTTTCAACTAATGTTGCAAATCTTTTTGGTGGAGAAGGTGTTCAAGCAGCTCCAGCTCAAGAGGGTGCACAACAGGAAGGAGTACAACCAGCTCCTGCTCAAGGTGGAGTTAGCTCTCCAATTAATGTTACAACTGCTATTGATGCTAATACATCACTTACGAAGATTGAGGATGCTATTAGAATGATAACTGATCAAAGAGC |
| *Borrelia* sp. | HNQW5 | ON060669 | *flaB* | TCTGATGATGCTGCTGGTATGGGTGTTGCTGGCAAGCTTAATTCTCAAATTAGGGGATTGTCTCAAGCTTCTAGAAATACTTCAAAGGCTATAAATTTTATTCAAACAACAGAAGGAAATTTGAATGAAGTAGAGAAGATATTAGTTAGAATGAAAGAGCTTGCTGTTCAGTCTGGTAATGGTACATACTCAGATGCAGACAGAGGTTCTATTCAAATTGAAATTGAACAACTTACAGATGAAATTAATAGAGTTGCTGATCAGGCTCAATATAACCAAATGCATATGCTATCCAACAAATCATCTGCTCAAAATGTAAAAACTGCTGAAGAGCTTGGAATGCAACCTGCAAAAATTAACACACCAGCATCACTAACTGGAGCACAAGCTTCATGGACATTGAGAGTTCAAGTAGGTGCAAATCAGGATGAAGCAATTGCTGTTAATATTTTTTCAACTAATGTTGCAAATCTTTTTGGTGGGGAAGGTGCGCAAGCTGCTCCAGTTCAAGAGGGTGCACAACAAGAAGGAGTTCAACCAGCTCCAGCTCAAGGTGGAGTTAATTCTCCAATTAATGTTACAACTGCTATTGATGCTAATACATCCCTTACAAAGATTGAAGATGCTATTAGAATGATAACTGATCAAAGAGC |
| *Borrelia* sp. | SDJN171 | ON060670 | *flaB* | TCTGATGATGCTGCTGGTATGGGTGTTGCTGGCAAACTTAATTCTCAAATTAGGGGATTGTCTCAAGCTTCTAGAAATACTTCAAAGGCTATAAATTTTATTCAAACAACAGAAGGAAATTTGAATGAAGTAGAGAAGATATTAGTTAGAATGAAAGAGCTTGCTGTTCAGTCTGGTAATGGTACATACTCAGATGCAGACAGAGGTTCTATTCAAATTGAAATTGAACAACTTACAGATGAAATTAATAGAGTTGCTGATCAGGCTCAATATAACCAAATGCATATGCTATCTAACAAATCATCTGCTCAAAGTGTGAAAACTGCTGAAGAGCTTGGAATGCAACCTGCAAAAATTAACACACCAGCATCACTAACTGGAGCACAAGCTTCATGGACATTGAGAGTTCAAGTAGGTGCAAATCAGGATGAAGCAATTGCTGTTAATATTTTTTCAACTAATGTTGCAAATCTTTTTGGTGGGGAAGGTGCGCAAGCTGCTCCAGTTCAAGAGGGTGCACAACAAGAAGGAGTTCAACCAGCTCCAGCTCAAGGTGGAGTTAATTCTCCAATTAATGTTACAACTGCTATTGATGCTAATACATCCCTTACAAAGATTGAAGATGCTATTAGAATGATAACTGATCAAAGAGC |
| *Borrelia miyamotoi* | NMG10G5 | ON060671 | *flaB* | TCTGATGATGCTGCTGGTATGGGTGTTGCTGGTAAGCTTAATTCACAAATTAGAGGATTGTCTCAAGCTTCTAGAAATACCTCAAAGGCTATAAATTTTATTCAAACAACAGAAGGAAATTTGAACGAGGTAGAGAAAGTATTAGTAAGAATGAAAGAACTTGCTGTTCAGTCTGGTAATGGTACATACTCAGATTCAGATAGAGGGTCTATTCAGATTGAAATTGAACAACTTACAGATGAAATAAACAGAATTGCTGATCAGGCTCAATACAACCAAATGCATATGTTATCTAATAAGTCAGCTGCTCAAAATGTAAAAACTGCTGAAGAGCTTGGAATGCAACCTGCAAAAATTAACACACCAGCATCATTGGCTGGATCACAAGCTTCATGGACATTGAGAGTGCATGTAGGTGCAAATCAGGATGAAGCAATTGCTGTCAATATTTATGCAGCTAATGTTGCAAATCTTTTTAATGGAGAAGGTGCTCAAGCAGCTCCAGCTCAAGAGGGAGCACAACAGGAGGGAGTTCAAGCAGTTCCAGCTCCAGCAGCCGCTCCAGTGCAAGGTGGAGTTAATTCTCCAATTAATGTTACAACTGCTATTGATGCTAATATGTCACTTTCAAAGATCGAAGATGCTATTAGAATGATAACTGATCAAAGAGC |
| *Borrelia theileri* | YN1F2 | ON113496 | *flaB* | TCTGATGATGCTGCTGGTATGGGTGTTGCTGGTAAACTTAATTCTCAAATTAGGGGATTATCTCAAGCTTCTAGAAATACTTCAAAGGCTATAAATTTTATTCAAACAACAGAAGGAAATTTGAATGAGGTGGAGAAGATATTAGTTAGAATGAAAGAACTTGCTGTTCAATCTGGTAATGGTACATACTCAGATGCAGACAGAGGTTCTATTCAAATTGAAATTGAACAACTTACAGATGAAATTAACAGAGTTGCTGATCAGGCTCAATATAACCAGATGCACATGTTATCTAACAAGTCATCTGCTCAAAATGTAAGAACTGCTGAAGAGCTTGGAATGCAACCTGCAAAAATTAATACACCAGCATCACTAACTGGATCACAAGCTTCATGGACATTGAGAGTTCAAGTAGGTGCAAATCAGGATGAAGCAATTGCTGTTAATATTTTTTCAACTAATGTCGCAAATCTTTTTGGTGGAGAAGGTGCTCAAGCAGCTCCAG |
| *Borrelia theileri* | YN3G7 | ON113497 | *flaB* | TCTGATGATGCTGCTGGTATGGGTGTTGCTGGTAAACTTAATTCTCAAATTAGGGGATTATCTCAAGCTTCTAGAAATACTTCAAAGGCTATAAATTTTATTCAAACAACAGAAGGAAATTTGAATGAGGTGGAGAAGATATTAGTTAGAATGAAAGAACTTGCTGTTCAATCTGGTAATGGTACATACTCAGATGCAGACAGAGGTTCTATTCAAATTGAAATTGAACAACTTACAGATGAAATTAACAGAGTTGCTGATCAGGCTCAATATAACCAGATGCACATGTTATCTAACAAGTCATCTGCTCAAAATGTAAGAACTGCTGAAGAGCTTGGAATGCAACCTGCAAAAATTAATACACCAGCATCACTAACTGGATCACAAGCTTCATGGACATTGAGAGTTCAAGTAGGTGCAAATCAGGATGAAGCAATTGCTGTTAATATTTTTTCAACTAATGTCGCAAATCTTTTTGGTGGAGAAGGTGCTCAAGCAGCTCCAG |
| *Borrelia theileri* | GD4E5 | ON184022 | *flaB* | GCTTCTGATGATGCTGCTGGTATGGGTGTTGCTGGTAAGCTTAATTCTCAAATTAGGGGATTATCTCAAGCTTCTAGAAATACTTCAAAGGCTATAAATTTTATTCAAACAACAGAAGGAAATTTGAATGAGGTGGAGAAGATATTAGTTAGAATGAAAGAACTTGCTGTTCAATCTGGTAATGGTACATACTCAGATGCAGACAGAGGTTCTATTCAAATTGAAATTGAACAACTTACAGATGAAATTAACAGAGTTGCTGATCAGGCTCAATATAACCAGATGCACATGTTATCTAACAAGTCATCTGCTCAAAATGTAAGAACTGCTGAAGAGCTTGGAATGCAACCTGCAAAAATTAATACACCAGCATCACTAACTGGATCACAAGCTTCATGGACATTGAGAGTTCAAGTAGGTGCAAATCAAGATGAAGCAATTGCTGTTAATATTTTTTCAACTAATGTTGCAAATCTTTTTGGTGGAGAAGGTGTTCAAGCAGCTCCAGCTCAAGAGGGTGCACAACAGGAAGGAGTACAACCAGCTCCTGCTCAAGGTGGAGTTAGCTCTCCAATTAATGTTACAACTGCTATTGATGCTAATACATCACTTACGAAGATTGAGGATGCTATTAGAATGATAACTGATCAAAGAGC |
| *Borrelia miyamotoi* | SD6B1 | ON184023 | *flaB* | GTGCATCTGATGATGCTGCTGGTATGGGTGTTGCTGGTAAGCTTAATTCACAAATTAGAGGATTGTCTCAAGCTTCTAGAAATACCTCAAAGGCTATAAATTTTATTCAAACAACAGAAGGAAATTTGAACGAGGTAGAGAAAGTATTAGTAAGAATGAAAGAACTTGCTGTTCAGTCTGGTAATGGTACATACTCAGATTCAGATAGAGGGTCTATTCAGATTGAAATTGAACAACTTACAGATGAAATAAACAGAATTGCTGATCAGGCTCAATACAACCAAATGCATATGTTATCTAATAAGTCAGCTGCTCAAAATGTAAAAACTGCTGAAGAGCTTGGAATGCAACCTGCAAAAATTAACACACCAGCATCATTGGCTGGATCACAAGCTTCATGGACATTGAGAGTGCATGTAGGTGCAAATCAGGATGAAGCAATTGCTGTCAATATTTATGCAGCTAATGTTGCAAATCTTTTTAATGGAGAAGGTGCTCAAGCAGCTCCAG |
| *Borrelia* sp. | HLJ6G11 | ON184024 | *flaB* | TGCGCTTCTGATGATGCTGCTGGTATGGGTGTTGCTGGTAAGCTTAATTCTCAAATTAGGGGATTATCTCAAGCTTCTAGAAATACTTCAAAGGCTATAAATTTTATTCAAACAACAGAAGGAAATTTGAATGAAGTAGAGAAGATATTAGTTAGAATGAAAGAACTTGCTGTTCAGTCTGGTAATGGTACATACTCAGATGCAGACAGAGGTTCTATTCAAATTGAAATTGAACAACTTACAGATGAAATTAACAGAGTGGCTGATCAGGCTCAATATAACCAGATGCATATGTTATCTAACAAGTCATCTGCTCAAAATGTAAAAACTGCTGAAGAGCTTGGAATGCAACCTGCAAAAATTAACACACCAGCATCACTAACTGGAGCACAAGCTTCATGGACATTAAGAGTTCAAGTGGGTGCAAATCAGGATGAAGCAATTGCAGTTAATATTTTTTCAACTAATGTTGCAAATCTTTTTGGTGGAGAAGGTGCTCAAGCAGCTCCAG |
| *Borrelia* sp. | HLJ19H7 | ON184025 | *flaB* | TGCGCTTCTGATGATGCTGCTGGTATGGGTGTTGCTGGTAAGCTTAATTCTCAAATTAGGGGATTATCTCAAGCTTCTAGAAATACTTCAAAGGCTATAAATTTTATTCAAACAACAGAAGGAAATTTGAATGAAGTAGAGAAGATATTAGTTAGAATGAAAGAACTTGCTGTTCAGTCTGGTAATGGTACATACTCAGATGCAGACAGAGGTTCTATTCAAATTGAAATTGAACAACTTACAGATGAAATTAACAGAGTGGCTGATCAGGCTCAATATAACCAGATGCATATGTTATCTAACAAGTCATCTGCTCAAAATGTAAAAACTGCTGAAGAGCTTGGAATGCAACCTGCAAAAATTAACACACCAGCATCACTAACTGGAGCACAAGCTTCATGGACATTAAGAGTTCAAGTGGGTGCAAATCAGGATGAAGCAATTGCAGTTAATATTTTTTCAACTAATGTTGCAAATCTTTTTGGTGGAGAAGGTGCTCAAGCAGCTCCAG |
| *Borrelia* sp. | NMG2D7 | ON184026 | *flaB* | TCTGATGATGCTGCTGGTATGGGTGTTGCTGGTAAGCTTAATTCTCAAATTAGGGGATTATCTCAAGCTTCTAGAAATACTTCAAAGGCTATAAATTTTATTCAAACAACAGAAGGAAATTTGAATGAAGTAGAGAAGATATTAGTTAGAATGAAAGAACTTGCTGTTCAGTCTGGTAATGGTACATACTCAGATGCAGACAGAGGTTCTATTCAAATTGAAATTGAACAACTTACAGATGAAATTAACAGAGTGGCTGATCAGGCTCAATATAACCAGATGCATATGTTATCTAACAAATCATCTGCTCAAAATGTAAAAACTGCTGAAGAGCTTGGAATGCAACCTGCAAAAATTAACACACCAGCATCACTAACTGGAGCACAAGCTTCATGGACATTAAGAGTTCAAGTGGGTGCAAATCAGGATGAAGCAATTGCAGTTAATATTTTTGCAACTAATGTTGCAAATCTTTTTGGTGGAGAAGGTGCTCAAGCAGCTCCAG |
| *Borrelia* sp. | NMG9B3 | ON184027 | *flaB* | TCTGATGATGCTGCTGGTATGGGTGTTGCTGGTAAGCTTAATTCTCAAATTAGGGGATTATCTCAAGCTTCTAGAAATACTTCAAAGGCTATAAATTTTATTCAAACAACAGAAGGAAATTTGAATGAAGTAGAGAAGATATTAGTTAGAATGAAAGAACTTGCTGTTCAGTCTGGTAATGGTACATACTCAGATGCAGACAGAGGTTCTATTCAAATTGAAATTGAACAACTTACAGATGAAATTAACAGAGTGGCTGATCAGGCTCAATATAACCAGATGCATATGTTATCTAACAAATCATCTGCTCAAAATGTAAAAACTGCTGAAGAGCTTGGAATGCAACCTGCAAAAATTAACACACCAGCATCACTAACTGGAGCACAAGCTTCATGGACATTAAGAGTTCAAGTGGGTGCAAATCAGGATGAAGCAATTGCAGTTAATATTTTTGCAACTAATGTTGCAAATCTTTTTGGTGGAGAAGGTGTTCAAGCAGCTCCAGCTCAAGAGGGTGCACAACAGGAAGGAGTTCAACCAGCTCCAGCTCAAGGTGGAGTTAGCTCTCCTATTAATGTTACAACTGCTATTGATGCTAATACATCACTTACAAAGATTGAAGATGCTATTAGAATGATAACTGATCAAAGAGC |
| *Borrelia* sp. | NMG9D11 | ON184028 | *flaB* | TCTGATGATGCTGCTGGTATGGGTGTTGCTGGTAAGCTTAATTCTCAAATTAGGGGATTATCTCAAGCTTCTAGAAATACTTCAAAGGCTATAAATTTTATTCAAACAACAGAAGGAAATTTGAATGAAGTAGAGAAGATATTAGTTAGAATGAAAGAACTTGCTGTTCAGTCTGGTAATGGTACATACTCAGATGCAGACAGAGGTTCTATTCAAATTGAAATTGAACAACTTACAGATGAAATTAACAGAGTGGCTGATCAGGCTCAATATAACCAGATGCATATGTTATCTAACAAATCATCTGCTCAAAATGTAAAAACTGCTGAAGAGCTTGGAATGCAACCTGCAAAAATTAACACACCAGCATCACTAACTGGAGCACAAGCTTCATGGACATTAAGAGTTCAAGTGGGTGCAAATCAGGATGAAGCAATTGCAGTTAATATTTTTGCAACTAATGTTGCAAATCTTTTTGGTGGAGAAGGTGTTCAAGCAGCTCCAGCTCAAGAGGGTGCACAACAGGAAGGAGTTCAACCAGCTCCAGCTCAAGGTGGAGTTAGCTCTCCTATTAATGTTACAACTGCTATTGATGCTAATACATCACTTACAAAGATTGAAGATGCTATTAGAATGATAACTGATCAAAGAGC |
| *Borrelia* sp. | NMG9F5 | ON184029 | *flaB* | GCATCTGATGATGCTGCTGGTATGGGTGTTGCTGGTAAGCTTAATTCTCAAATTAGGGGATTATCTCAAGCTTCTAGAAATACTTCAAAGGCTATAAATTTTATTCAAACAACAGAAGGAAATTTGAATGAAGTAGAGAAGATATTAGTTAGAATGAAAGAACTTGCTGTTCAGTCTGGTAATGGTACATACTCAGATGCAGACAGAGGTTCTATTCAAATTGAAATTGAACAACTTACAGATGAAATTAACAGAGTGGCTGATCAGGCTCAATATAACCAGATGCATATGTTATCTAACAAATCATCTGCTCAAAATGTAAAAACTGCTGAAGAGCTTGGAATGCAACCTGCAAAAATTAACACACCAGCATCACTAACTGGAGCACAAGCTTCATGGACATTAAGAGTTCAAGTGGGTGCAAATCAGGATGAAGCAATTGCAGTTAATATTTTTGCAACTAATGTTGCAAATCTTTTTGGTGGAGAAGGTGCTCAAGCAGCTCCAG |
| *Borrelia persica* | XJAL047 | ON361163 | *flaB* | TAATACGTCAGCCATAAATGCTTCAAGAAATAATGCTATTAATGTTGCTAATCTTAGCAAAACTCAAGAAAAACTTTCTAGTGGGTATAGAATTAATCGTGCATCTGATGATGCTGCTGGTATGGGTGTTGCTGGGAAAATTAATGCTCAAATTAGGGGATTATCTCAAGCTTCTCGCAATACTTCAAAGGCTATAAATTTTATTCAAACAACAGAAGGGAATTTAAATGAAGTAGAACAAATACTAGTAAGAATGAAAGAACTTGCTGTTCAATCTGGTAATGGTACATATTCAGATGCGGATAGAGGTTCTATTCAAATTGAAATTGAGCAACTTACAGATGAAATTAATAGAATTGCTGATCAGGCGCAATATAACCAAATGCATATGTTGTCTAATAAATCAGCTGCTGATAATGTAAGGACAGCTGAAGAACTTGGAATGCAACCTGCAAAAATTAATACACCAACCTCATTAACCGGGTCACAAGCTTCATGGACATTAAGAGTACATGTGGGTGCAAATCAAGATGAAGCAATTGCTATTAATATTTATGCAGCTAATGTTTCAAGTCTTTTCTCAGGTGAGGGTGCTCAACAAGCAACTCAAAATCAAGAAGGAGTACAGCAGCCAGCAGCAGCTCCAACTCAAGGTGGGATTAATTCTCCAGTTAATGTTACAACTGCTGTTGATGCCAATGTTTCACTTACAAAAATAGAAGATGCTATTAGAATGATAACTGATCAAAGAGC |
| *Borrelia persica* | XJAL048 | ON361164 | *flaB* | TAATACGTCAGCCATAAATGCTTCAAGAAATAATGCTATTAATGTTGCTAATCTTAGCAAAACTCAAGAAAAACTTTCTAGTGGGTATAGAATTAATCGTGCATCTGATGATGCTGCTGGTATGGGTGTTGCTGGGAAAATTAATGCTCAAATTAGGGGATTATCTCAAGCTTCTCGCAATACTTCAAAGGCTATAAATTTTATTCAAACAACAGAAGGGAATTTAAATGAAGTAGAACAAATACTAGTAAGAATGAAAGAACTTGCTGTTCAATCTGGTAATGGTACATATTCAGATGCGGATAGAGGTTCTATTCAAATTGAAATTGAGCAACTTACAGATGAAATTAATAGAATTGCTGATCAGGCGCAATATAACCAAATGCATATGTTGTCTAATAAATCAGCTGCTGATAATGTAAGGACAGCTGAAGAACTTGGAATGCAACCTGCAAAAATTAATACACCAACCTCATTAACCGGGTCACAAGCTTCATGGACATTAAGAGTACATGTGGGTGCAAATCAAGATGAAGCAATTGCTATTAATATTTATGCAGCTAATGTTTCAAGTCTTTTCTCAGGTGAGGGTGCTCAACAAGCAACTCAAAATCAAGAAGGAGTACAGCAGCCAGCAGCAGCTCCAACTCAAGGTGGGATTAATTCTCCAGTTAATGTTACAACTGCTGTTGATGCCAATGTTTCACTTACAAAAATAGAAGATGCTATTAGAATGATAACTGATCAAAGAGC |
| *Borrelia persica* | XJDLT014 | ON361165 | *flaB* | TAATACGTCAGCCATAAATGCTTCAAGAAATAATGCTATTAATGTTGCTAATCTTAGCAAAACTCAAGAAAAACTTTCTAGTGGGTATAGAATTAATCGTGCATCTGATGATGCTGCTGGTATGGGTGTTGCTGGGAAAATTAATGCTCAAATTAGGGGATTATCTCAAGCTTCTCGCAATACTTCAAAGGCTATAAATTTTATTCAAACAACAGAAGGGAATTTAAATGAAGTAGAACAAATACTAGTAAGAATGAAAGAACTTGCTGTTCAATCTGGTAATGGTACATATTCAGATGCGGATAGAGGTTCTATTCAAATTGAAATTGAGCAACTTACAGATGAAATTAATAGAATTGCTGATCAGGCGCAATATAACCAAATGCATATGTTGTCTAATAAATCAGCTGCTGATAATGTAAGGACAGCTGAAGAACTTGGAATGCAACCTGCAAAAATTAATACACCAACCTCATTAACCGGGTCACAAGCTTCATGGACATTAAGAGTACATGTGGGTGCAAATCAAGATGAAGCAATTGCTATTAATATTTATGCAGCTAATGTTTCAAGTCTTTTCTCAGGTGAGGGTGCTCAACAAGCAACTCAAAATCAAGAAGGAGTACAGCAGCCAGCAGCAGCTCCAACTCAAGGTGGGATTAATTCTCCAGTTAATGTTACAACTGCTGTTGATGCCAATGTTTCACTTACAAAAATAGAAGATGCTATTAGAATGATAACTGATCAAAGAGC |
| *Borrelia persica* | XJDLT021 | ON361166 | *flaB* | TAATACGTCAGCCATAAATGCTTCAAGAAATAATGCTATTAATGTTGCTAATCTTAGCAAAACTCAAGAAAAACTTTCTAGTGGGTATAGAATTAATCGTGCATCTGATGATGCTGCTGGTATGGGTGTTGCTGGGAAAATTAATGCTCAAATTAGGGGATTATCTCAAGCTTCTCGCAATACTTCAAAGGCTATAAATTTTATTCAAACAACAGAAGGGAATTTAAATGAAGTAGAACAAATACTAGTAAGAATGAAAGAACTTGCTGTTCAATCTGGTAATGGTACATATTCAGATGCGGATAGAGGTTCTATTCAAATTGAAATTGAGCAACTTACAGATGAAATTAATAGAATTGCTGATCAGGCGCAATATAACCAAATGCATATGTTATCTAATAAATCAGCTTCTGATAATGTAAAGACAGCTGAAGAACTTGGAATGCAACCTGCAAAAATTAATACACCAACCTCATTAACCGGGTCACAAGCTTCATGGACATTAAGAGTACATGTGGGTGCAAATCAAGATGAAGCAATTGCTATTAATATTTATGCAGCTAATGTTTCAAGTCTTTTCTCAGGTGAGGGTGCTCAACAAGCAACTCAAAATCAAGAAGGAGTACAACAGCCAGCAGCAGCTCCAACTCAAGGTGGGATTAATTCTCCAGTTAATGTTACAACTGCTGTTGATGCCAATGTTTCACTTACAAAAATAGAAGATGCTATTAGAATGATAACTGATCAAAGAGC |
| *Borrelia persica* | XJAL047 | ON361167 | *glpQ* | ATAGCTCACAGAGGTGCAAGCGGATATTTGCCAGAACATACCCTAGAATCCAAAGCATACGCTCATGCATTAGGAGCTGATTACATAGAACAAGATATTGTTTTAACAAAAGATAATATTCCTATCATCATGCACGATCCAGAAATAGATACAACAACAAATGTTGCCAAACTATTTCCAGACAGAGCTAGAGAAAATGGAAGATATTATTCTGTTGATTTCACATTAGAAGAAATTAAATCATTAAGCCTTAGTGAAAGATTCGACCCAGAAACAAAAAAACCAATATATCCTAATCGTTTCCCTGTAACTGGATATGATTTTAAAATTCCAACCTTAGAGGAAGAACTACAATTCATACAAGGATTAAACAAAAGTACAGGAAAGAATATTGGAATTTACCCTGAAATCAAAAAACCTTTTTGGCACAAACAACAGGGCAAAGATATATCCAAAATAGTAATAGAAATTTTAAATAAATATGGATATAAATCAAAAGAAGACAAGATTTATCTTCAAACATTCGATTTTGACGAAATGAAGAGAATAAGAGAAGAACTTGGATATCAAGGAAAATTAATAATGCTCATTGGAGAAAATGATTGGGAAGAAGCACCAACAGATTATGAATACATTAAATCAGAAGAAGGTATGGCTGAAATTGCAAAATATGCTGACGGAATTGGACCTTGGATACCTCAAATCATAATCAATGGAGAAATCACAGGTCTTACAAGTTTAGCACACAAATATAACATGAAAGTTCATGCTTATACATTTAGAACTGATGCATTGCCTTCATATGTAAAA |
| *Borrelia persica* | XJAL048 | ON361168 | *glpQ* | ATAGCTCACAGAGGTGCAAGCGGATATTTGCCAGAACATACCCTAGAATCCAAAGCATACGCTCATGCATTAGGAGCTGATTACATAGAACAAGATATTGTTTTAACAAAAGATAATATTCCTATCATCATGCACGATCCAGAAATAGATACAACAACAAATGTTGCCAAACTATTTCCAGACAGAGCTAGAGAAAATGGAAGATATTATTCTGTTGATTTCACATTAGAAGAAATTAAATCATTAAGCCTTAGTGAAAGATTCGACCCAGAAACAAAAAAACCAATATATCCTAATCGTTTCCCTGTAACTGGATATGATTTTAAAATTCCAACCTTAGAGGAAGAACTACAATTCATACAAGGATTAAACAAAAGTACAGGAAAGAATATTGGAATTTACCCTGAAATCAAAAAACCTTTTTGGCACAAACAACAGGGCAAAGATATATCCAAAATAGTAATAGAAATTTTAAATAAATATGGATATAAATCAAAAGAAGACAAGATTTATCTTCAAACATTCGATTTTGACGAAATGAAGAGAATAAGAGAAGAACTTGGATATCAAGGAAAATTAATAATGCTCATTGGAGAAAATGATTGGGAAGAAGCACCAACAGATTATGAATACATTAAATCAGAAGAAGGTATGGCTGAAATTGCAAAATATGCTGACGGAATTGGACCTTGGATACCTCAAATCATAATCAATGGAGAAATCACAGGTCTTACAAGTTTAGCACACAAATATAACATGAAAGTTCATGCTTATACATTTAGAACTGATGCATTGCCTTCATATGTAAAA |
| *Borrelia persica* | XJDLT014 | ON361169 | *glpQ* | ATAGCTCACAGAGGTGCAAGCGGATATTTGCCAGAACATACCCTAGAATCCAAGGCATACGCTCATGCATTAGGAGCTGATTACATAGAACAAGATATTGTTTTAACAAAAGATAATATTCCTATCATCATGCACGATCCAGAAATAGATACAACAACAAATGTTGCCAAAATATTTCCAGACAGAGCTAGAGAAAATGGAAGATATTATTCTGTTGATTTCACATTAGAAGAAATCAAATCATTAAGCCTTAGTGAAAGATTCGACCCAGAAACAAAAAAACCAATATATCCTAATCGTTTCCCTGTAACTGGATATGATTTTAAAATTCCAACCTTAGAGGAAGAACTACAATTCATACAAGGATTAAACAAAAGCACAGGAAAGAATATTGGAATTTACCCTGAAATCAAAAAACCTTTTTGGCACAAACAACAGGGCAAAGATATATCCAAAATAGTAATAGAAATTTTAAATAAATATGGATATAAATCAAAAGAAGATAAGATTTATCTTCAAACATTCGATTTTGACGAAATGAAGAGAATAAGAGAAGAACTTGGATATCAAGGAAAATTAATAATGCTCATTGGAGAAAATGATTGGGAAGAAGCACCAACAGATTATGAATACATTAAGTCAGAAGAAGGTATGGCTGAAATTGCAAAATATGCTGACGGAATTGGACCTTGGATACCTCAAATCATAATCAATGGAGAAATCACAGGTCTTACAAGTTTAGCACACAAATATAACATGAAAGTTCATGCTTATACATTTAGAACTGATGCATTGCCTTCATATGTAAAA |
| *Borrelia persica* | XJAL009 | ON361170 | *glpQ* | ATAGCTCACAGAGGTGCAAGCGGATATTTGCCAGAACATACCCTAGAATCCAAAGCATACGCTCATGCATTAGGAGCTGATTACATAGAACAAGATATTGTTTTAACAAAAGATAATATTCCTATCATCATGCACGATCCAGAAATAGATACAACAACAAATGTTGCCAAACTATTTCCAGACAGAGCTAGAGAAAATGGAAGATATTATTCTGTTGATTTCACATTAGAAGAAATTAAATCATTAAGCCTTAGTGAAAGATTCGACCCAGAAACAAAAAAACCAATATATCCTAATCGTTTCCCTGTAACTGGATATGATTTTAAAATTCCAACCTTAGAGGAAGAACTACAATTCATACAAGGATTAAACAAAAGCACAGGAAAGAATATTGGAATTTACCCTGAAATCAAAAAACCTTTTTGGCACAAACAACAGGGCAAAGATATATCCAAAATAGTAATAGAAATTTTAAATAAATATGGATATAAATCAAAAGAAGATAAAATTTATCTTCAAACATTCGATTTTGACGAAATGAAGAGAATAAGAGAAGAACTTGGATATCAAGGAAAATTAATAATGCTCATTGGAGAAAATGATTGGGAAGAAGCACCAACAGATTATGAATACATTAAATCAGAAGAAGGTATGGCTGAAATTGCAAAATATGCTGACGGAATTGGACCTTGGATACCTCAAATCATAATCAATGGAGAAATCACAGGTCTTACAAGTTTAGCACACAAATATAACATGAAAGTTCATGCTTATACATTTAGAACTGATGCATTGCCTTCATATGTAAAA |
| *Borrelia miyamotoi* | HLJ7E3 | ON148112 | *glpQ* | ATGGGTTCAAACAAAAAGTCACCATTGATCATAGCTCACAGGGGTGCTAGTGGGTATCTTCCAGAACATACCTTAGAAGCTAAAGCATATGCTTATGCATTAGGAGCTGATTATCTAGAACAAGACATAGTTCTAACAAAGGACAATATTCCTGTTATAATGCACGACCCAGAAATTGACACAACCACAAATGTTGCACAATTATTTCCCAATCGAGCTAGAGAAAACGGACGATATTACGCCACTGACTTCACACTAACTGAACTTAAATCACTAAGTCTCAGTGAAAGATTTGATCCTGAAAACAAAAAACCAATATACCCTAATCGTTTCCCCTTAAATGAATATAATTTTAAAATTCCAACTTTAGAAGAAGAAATAAAATTCATACAAGGACTAAATAAAAGCACAGGAAGAAATGTTGGGATTTACCCTGAAATTAAAAAACCCTTCTGGCATAAACAACAAGGTAAAGACATC |
| *Borrelia* sp. | NMG9F5 | ON148113 | *glpQ* | CATACGCTTATGCCTTAGGCGCTGATTATTTAGAACAAGACATAGTGCTAACAAAGGACAATATTCCTGTTATAATGCATGATCCAGAACTTGACACAACCACAAATGTTAAACAACTATTTCCTAACCGAGCTAGAGAAAACGGGCGATATTACTCTACCGACTTTACACTAACTGAACTTAAATCACTAAGTGTTAGTGAAAGATTTAATCCTGAAAATAAACAACCAATATATCCTAGCCGTTTTCCACTAAATGGATACAATTTTAAAATTGTAACATTAGAAGAAGAAATACAATTTATACAAGGACTAAATAAAAGCACAGGAAAAAATGTCGGAATTTACCCTGAAATTAAAAAGCCCTTCTGGCATAAACAACAAGGTAAAGACATC |
| *Borrelia* sp. | SDJN171 | ON148114 | *glpQ* | CTTGTTGTTTATGCCAGAAGGGTTTTTTAATTTCAGGGTAGATTCCAACATTTTTTCCTGTGCTTTTATTTAGTCCTTGTATAAATTGTATTTCTTCTTCTAAAGTTGGAATTTTAAAATTGCACTCAGTTTGTGGGAAACGGTTGGAATATATTGGTTGTTTATTTTCAGGATCAAATCTTTCACTAAGATTTAGTAATTTAATTTCGGCTAGTGTAAAGTCAGTAGAGTAATATTGTCCGTCTTCTCTAGCTCGGTTAGGAAATAGTTGTTTAACATTTGTGGTTGTATTAAGTGTTGGATCGTGCATTATAATAGGAATATTGTCTTTTGTTAACACTATGTCTTGTTCTAAATAATCAGCTCCTAAAGCATAAGCGTATG |
| *Borrelia theileri* | HN1F2 | ON148115 | *glpQ* | CATACGCTTATGCCTTAGGCGCTGATTATTTAGAACAAGACATAGTACTAACAAAGGACAATATTCCTATTATAATTCACGATCCAGAGCTTAATACAACCACAAATGTTAAACAACTATTTCCTAACCGAGCCAGAGAAGACGGACAATATTACTCTACTGACTTTACAATAGCCGAAATTAAATTGTTAAGTCTTAGTGAAAGATTTAATCCTGAAAATAAACAACCAATATATCCTAACCGTTTTCCACTAACTAAATACAATTTTAAAATTCCAACTTTAGAAGAAGAAATACAATTTATACAAGGACTAAATAAAAGCACAGGAAAAAATGTTGGAATCTACCCTGAAATTAAAAAACCCTTTTGGCATAAACAACAAGGTAAAGACATC |
| *Borrelia theileri* | GD4E5 | ON148116 | *glpQ* | CATACGCTTATGCTTTAGGAGCTGATTATTTAGAACAAGACATAGTACTAACAAAGGACAATATTCCTATTATAATTCACGATCCAGAGCTTAATACAACCACAAATGTTAAACAACTATTTCCTAACCGAGCCAGAGAAGACGGACAATATTACTCTACTGACTTTACAATAGCCGAAATTAAATTGTTAAGTCTTAGTGAAAGATTTAATCCTGAAAATAAACAACCAATATATCCTAACCGTTTTCCACTAACTAAATACAATTTTAAAATTCCAACTTTAGAAGAAGAAATACAATTTATACAAGGACTAAATAAAAGCACAGGAAAAAATGTTGGAATCTACCCTGAAATTAAAAAACCCTTTTGGCATAAACAACAAGGTAAAGACATC |
| *Borrelia theileri* | GD4C9 | ON148117 | *glpQ* | GATGTCTTTACCTTGTTGTTTATGCCAAAAGGGTTTTTTAATTTCAGGGTAGATTCCAACATTTTTTCCTGTGCTTTTATTTAGTCCTTGTATAAATTGTATTTCTTCTTCTAAAGTTGGAATTTTAAAATTGTATTTAGTTAGTGGAAAACGGTTAGGATATATTGGTTGTTTATTTTCAGGATTAAATCTTTCACTAAGACTTAACAATTTAATTTCGGCTATTGTAAAGTCAGTAGAGTAATATTGTCCGTCTTCTCTGGCTCGGTTAGGAAATAGTTGTTTAACATTTGTGGTTGTATTAAGCTCTGGATCGTGAATTATAATAGGAATATTGTCCTTTGTTAGTACTATGTCTTGTTCTAAATAATCAGCTCCTAAAGCATAAGCGTATG |
| *Borrelia miyamotoi* | SD6B1 | ON148118 | *glpQ* | TGTCTTTTTTCCTTGTTGTTTATGCCAGAAGGGTTTTTTAATTTCAGGGTAAATCCCAACATTTCTTCCTGTGCTTTTATTTAGTCCTTGTATGAATTTTATTTCTTCTTCTAAAGTTGGAATTTTAAAATTATATTCATTTAAGGGGAAACGATTAGGGTATATTGGTTTTTTGTTTTCAGGATCAAATCTTTCACTGAGACTTAGTGATTTAAGTTCAGTTAGTGTGAAGTCAGTGGCGTAATATCGTCCGTTTTCTCTAGCTCGATTGGGAAATAATTGTGCAACATTTGTGGTTGTGTCAATTTCTGGGTCGTGCATTATAACAGGAATATTGTCCTTTGTTAGAACTATGTCTTGTTCTAGATAATCAGCTCCTAATGCATAAGCATATG |
| *Borrelia miyamotoi* | HLJ6F5 | ON148119 | *glpQ* | TGTCTTTTTTCCTTGTTGTTTATGCCAGAAGGGTTTTTTAATTTCAGGGTAAATCCCAACATTTCTTCCTGTGCTTTTATTTAGTCCTTGTATGAATTTTATTTCTTCTTCTAAAGTTGGAATTTTAAAATTATATTCATTTAAGGGGAAACGATTAGGGTATATTGGTTTTTTGTTTTCAGGATCAAATCTTTCACTGAGACTTAGTGATTTAAGTTCAGTTAGTGTGAAGTCAGTGGCGTAATATCGTCCGTTTTCTCTAGCTCGATTGGGAAATAATTGTGCAACATTTGTGGTTGTGTCAATTTCTGGGTCGTGCATTATAACAGGAATATTGTCCTTTGTTAGAACTATGTCTTGTTCTAGATAATCAGCTCCTAATGCATAAGCATATG |
| *Borrelia* sp. | HLJ6G11 | ON148120 | *glpQ* | CATACGCTTATGCTTTAGGAGCTGATTATTTAGAACAAGACATAGTGCTAACAAAGGACAATATTCCTGTTATAATGCATGATCCAGAACTTGACACAACCACAAATGTTAAACAACTATTTCCTAACCGAGCTAGAGAAAACGGGCGATATTACTCTACCGACTTTACACTAACTGAACTTAAATCACTAAGTATTAGTGAAAGATTTAATCCTGAAAATCAAAAACCAATATATCCTAGCCGTTTTCCACTAAATGAATACAATTTTAAAATTCCAACATTAGAAGAAGAAATACAATTTATACAAGGACTAAATAAAAGCACAGGAAAAAATGTCGGAATTTACCCTGAAATTAAAAAACCCTTCTGGCATAAACAACAAGGTAAAGACATC |
| *Borrelia* sp. | HLJ9E4 | ON148121 | *glpQ* | CATACGCTTATGCCTTAGGAGCTGATTATTTAGAACAAGACATAGTGCTAACAAAGGACAATATTCCTGTTATAATGCATGATCCAGAACTTGACACAACCACAAATGTTAAACAACTATTTCCTAACCGAGCTAGAGAAAACGGGCGATATTACTCTACCGACTTTACACTAACTGAACTTAAATCACTAAGTATTAGTGAAAGATTTAATCCTGAAAATCAAAAACCAATATATCCTAGCCGTTTTCCACTAAATGAATACAATTTTAAAATTCCAACATTAGAAGAAGAAATACAATTTATACAAGGACTAAATAAAAGCACAGGAAAAAATGTCGGAATTTACCCTGAAATTAAAAAACCCTTCTGGCATAAACAACAAGGAAAAGACATC |
| *Borrelia* sp. | HLJ9C11 | ON148122 | *glpQ* | GATGTCTTTACCTTGTTGTTTATGCCAGAAGGGCTTTTTAATTTCAGGGTAAATTCCGACATTTTTTCCTGTGCTTTTATTTAGTCCTTGTATAAATTGTATTTCTTCTTCTAATGTTACAATTTTAAAATTGTATCCATTTAGTGGAAAACGGCTAGGATATATTGGTTGTTTATTTTCAGGATTAAATCTTTCACTAACACTTAGTGATTTAAGTTCAGTTAGTGTAAAGTCGGTAGAGTAATATCGCCCGTTTTCTCTAGCTCGGTTAGGAAATAGTTGTTTAACATTTGTGGTTGTGTCAAGTTCTGGATCATGCATTATAACAGGAATATTGTCCTTTGTTAGCACTATGTCTTGTTCTAAATAATCAGCTCCTAAAGCATAAGCGTATG |
| *Borrelia* sp. | HLJ19H7 | ON148123 | *glpQ* | CATACGCTTATGCCTTAGGCGCTGATTATTTAGAACAAGACATAGTGCTAACAAAGGACAATATTCCTGTTATAATGCATGATCCAGAACTTGACACAACCACAAATGTTAAACAACTATTTCCTAACCGAGCTAGAGAAAACGGGCGATATTACTCTACCGACTTTACACTAACTGAACTTAAATCACTAAGTATTAGTGAAAGATTTAATCCTGAAAATCAAAAACCAATATATCCTAGCCGTTTTCCACTAAATGAATACAATTTTAAAATTCCAACATTAGAAGAAGAAATACAATTTATACAAGGACTAAATAAAAGCACAGGAAAAAATGTCGGAATTTACCCTGAAATTAAAAAACCCTTCTGGCATAAACAACAAGGTAAAGACATC |
| *Borrelia* sp. | NMG9B3 | ON148124 | *glpQ* | GATGTCTTTACCTTGTTGTTTATGCCAGAAGGGCTTTTTAATTTCAGGGTAAATTCCGACATTTTTTCCTGTGCTTTTATTTAGTCCTTGTATAAATTGTATTTCTTCTTCTAATGTTACAATTTTAAAATTGTATCCATTTAGTGGAAAACGGCTAGGATATATTGGTTGTTTATTTTCAGGATTAAATCTTTCACTAACACTTAGTGATTTAAGTTCAGTTAGTGTAAAGTCGGTAGAGTAATATCGCCCGTTTTCTCTAGCTCGGTTAGGAAATAGTTGTTTAACATTTGTGGTTGTGTCAAGTTCTGGATCATGCATTATAACAGGAATATTGTCCTTTGTTAGCACTATGTCTTGTTCTAAATAATCAGCGCCCAAGGCATAAGCGTATG |
| *Borrelia* sp. | NMG9D11 | ON148125 | *glpQ* | GATGTCTTTACCTTGTTGTTTATGCCAGAAGGGCTTTTTAATTTCAGGGTAAATTCCGACATTTTTTCCTGTGCTTTTATTTAGTCCTTGTATAAATTGTATTTCTTCTTCTAATGTTACAATTTTAAAATTGTATCCATTTAGTGGAAAACGGCTAGGATATATTGGTTGTTTATTTTCAGGATTAAATCTTTCACTAACACTTAGTGATTTAAGTTCAGTTAGTGTAAAGTCGGTAGAGTAATATCGCCCGTTTTCTCTAGCTCGGTTAGGAAATAGTTGTTTAACATTTGTGGTTGTGTCAAGTTCTGGATCATGCATTATAACAGGAATATTGTCCTTTGTTAGCACTATGTCTTGTTCTAAATAATCAGCTCCTAAAGCATAAGCGTATG |
| *Borrelia* sp. | NMG10E7 | ON148126 | *glpQ* | GATGTCTTTACCTTGTTGTTTATGCCAGAAGGGCTTTTTAATTTCAGGGTAAATTCCGACATTTTTTCCTGTGCTTTTATTTAGTCCTTGTATAAATTGTATTTCTTCTTCTAATGTTACAATTTTAAAATTGTATCCATTTAGTGGAAAACGGCTAGGATATATTGGTTGTTTATTTTCAGGATTAAATCTTTCACTAACACTTAGTGATTTAAGTTCAGTTAGTGTAAAGTCGGTAGAGTAATATCGCCCGTTTTCTCTAGCTCGGTTAGGAAATAGTTGTTTAACATTTGTGGTTGTGTCAAGTTCTGGATCATGCATTATAACAGGAATATTGTCCTTTGTTAGCACTATGTCTTGTTCTAAATAATCAGCTCCTAAAGCATAAGCGTATG |
| *Borrelia miyamotoi* | NMG10G5 | ON148127 | *glpQ* | CATACGCTTATGCCTTAGGAGCTGATTATCTAGAACAAGACATAGTTCTAACAAAGGACAATATTCCTGTTATAATGCACGACCCAGAAATTGACACAACCACAAATGTTGCACAATTATTTCCCAATCGAGCTAGAGAAAACGGACGATATTACGCCACTGACTTCACACTAACTGAACTTAAATCACTAAGTCTCAGTGAAAGATTTGATCCTGAAAACAAAAAACCAATATACCCTAATCGTTTCCCCTTAAATGAATATAATTTTAAAATTCCAACTTTAGAAGAAGAAATAAAATTCATACAAGGACTAAATAAAAGCACAGGAAGAAATGTTGGGATTTACCCTGAAATTAAAAAACCCTTCTGGCATAAACAACAAGGTAAAGACATC |
| *Borrelia miyamotoi* | NMG10H11 | ON148128 | *glpQ* | GATGTCTTTACCTTGTTGTTTATGCCAGAAGGGCTTTTTAATTTCAGGGTAAATTCCGACATTTTTTCCTGTGCTTTTATTTAGTCCTTGTATAAATTGTATTTCTTCTTCTAATGTTACAATTTTAAAATTGTATCCATTTAGTGGAAAACGGCTAGGATATATTGGTTGTTTATTTTCAGGATTAAATCTTTCACTAACACTTAGTGATTTAAGTTCAGTTAGTGTAAAGTCGGTAGAGTAATATCGCCCGTTTTCTCTAGCTCGGTTAGGAAATAGTTGTTTAACATTTGTGGTTGTGTCAAGTTCTGGATCATGCATTATAACAGGAATATTGTCCTTTGTTAGCACTATGTCTTGTTCTAAATAATCAGCTCCTAAAGCATAAGCGTATG |
| *Borrelia theileri* | YN1B2 | ON148129 | *glpQ* | ATGGGTTCAAACAAAAAGTCACCATTAGTTATAGCTCACAGAGGTGCTAGTGGATACCTTCCAGAACATACTTTAGAATCTAAAGCATATGCTTATGCCTTAGGAGCTGATTATTTAGAACAAGACATAGTACTAACAAAGGACAATATTCCTATTATAATTCACGATCCAGAGCTTAATACAACCACAAATGTTAAACAACTATTTCCTAACCGAGCCAGAGAAGACGGACAATATTACTCTACTGACTTTACAATAGCCGAAATTAAATTGTTAAGTCTTAGTGAAAGATTTAATCCTGAAAATAAACAACCAATATATCCTAACCGTTTTCCACTAACTAAATACAATTTTAAAATTCCAACTTTAGAAGAAGAAATACAATTTATACAAGGACTAAATAAAAGCACAGGAAAAAATGTTGGAATCTACCCTGAAATTAAAAAACCCTTTTGGCATAAACAACAAGGTAAAGACATC |
| *Borrelia theileri* | YN1F2 | ON148130 | *glpQ* | ATGGGTTCAAACAAAAAGTCACCATTAGTTATAGCTCACAGAGGTGCTAGTGGATACCTTCCAGAACATACTTTAGAATCTAAAGCATATGCTTATGCCTTAGGAGCTGATTATTTAGAACAAGACATAGTACTAACAAAGGACAATATTCCTATTATAATTCACGATCCAGAGCTTAATACAACCACAAATGTTAAACAACTATTTCCTAACCGAGCCAGAGAAGACGGACAATATTACTCTACTGACTTTACAATAGCCGAAATTAAATTGTTAAGTCTTAGTGAAAGATTTAATCCTGAAAATAAACAACCAATATATCCTAACCGTTTTCCACTAACTAAATACAATTTTAAAATTCCAACTTTAGAAGAAGAAATACAATTTATACAAGGACTAAATAAAAGCACAGGAAAAAATGTTGGAATCTACCCTGAAATTAAAAAACCCTTTTGGCATAAACAACAAGGTAAAGACATC |
| *Borrelia theileri* | YN3G7 | ON148131 | *glpQ* | ATGGGTTCAAACAAAAAGTCACCATTAGTTATAGCTCACAGAGGTGCTAGTGGATACCTTCCAGAACATACTTTAGAATCTAAAGCATATGCTTATGCCTTAGGAGCTGATTATTTAGAACAAGACATAGTACTAACAAAGGACAATATTCCTATTATAATTCACGATCCAGAGCTTAATACAACCACAAATGTTAAACAACTATTTCCTAACCGAGCCAGAGAAGACGGACAATATTACTCTACTGACTTTACAATAGCCGAAATTAAATTGTTAAGTCTTAGTGAAAGATTTAATCCTGAAAATAAACAACCAATATATCCTAACCGTTTTCCACTAACTAAATACAATTTTAAAATTCCAACTTTAGAAGAAGAAATACAATTTATACAAGGACTAAATAAAAGCACAGGAAAAAATGTTGGAATCTACCCTGAAATTAAAAAACCCTTTTGGCATAAACAACAAGGTAAAGACATC |
| *Borrelia* sp. | HNQW5 | ON148132 | *glpQ* | CATACGCTTATGCTTTAGGAGCTGATTATTTAGAACAAGACATAGTGTTAACAAAAGACAATATTCCTATTATAATGCACGATCCAACACTTAATACAACCACAAATGTTAAACAACTATTTCCTAACCGAGCTAGAGAAGACGGACAATATTACTCTACTGACTTTACACTAGCCGAAATTAAATTACTAAATCTTAGTGAAAGATTTGATCCTGAAAATAAACAACCAATATATTCCAACCGTTTCCCACAAACTGAGTGCAATTTTAAAATTCCAACTTTAGAAGAAGAAATACAATTTATACAAGGACTAAATAAAAGCACAGGAAAAAATGTTGGAATCTACCCTGAAATTAAAAAACCCTTCTGGCATAAACAACAAGGTAAAGACATC |

**Table S4. Positive rate of relapsing fever *Borrelia* in ticks from six eco-climate regions in China***.

| **Tick species** | **Northeastern China** | **Inner Mongolia-Xinjiang** | **Northern** **China** | **Central China** | **Southern China** | **Southwestern China** | **Total** |
| --- | --- | --- | --- | --- | --- | --- | --- |
| *Ixodes persulcatus* |  |  |  |  |  |  |  |
| NPP/NTP/NTT | 27/218/930 | 6/19/95 |  |  |  |  | 33/237/1025 |
| Prevalence (%, 95% CI) | 3.06 (2.07-4.36) | 7.13 (3.00-14.46) |  |  |  |  | 3.42 (2.41-4.72) |
| *Ixodes ovatus* |  |  |  |  |  |  |  |
| NPP/NTP/NTT |  |  |  |  |  | 6/126/126 | 6/126/126 |
| Prevalence (%, 95% CI) |  |  |  |  |  | 4.76 (1.97-9.60) | 4.76 (1.97-9.60) |
| *Haemaphysalis japonica* |  |  |  |  |  |  |  |
| NPP/NTP/NTT | 6/70/224 |  |  |  |  |  | 6/70/224 |
| Prevalence (%, 95% CI) | 2.81 (1.16-5.79) |  |  |  |  |  | 2.81 (1.16-5.79) |
| *Haemaphysalis concin* |  |  |  |  |  |  |  |
| NPP/NTP/NTT | 2/38/101 | 48/931/2465 |  |  |  |  | 50/969/2566 |
| Prevalence (%, 95% CI) | 2.02 (0.36-6.52) | 2.13 (1.59-2.81) |  |  |  |  | 2.13 (1.60-2.79) |
| *Haemaphysalis longicornis* |  |  |  |  |  |  |  |
| NPP/NTP/NTT | 12/516/1982 |  | 13/969/2017 |  |  |  | 25/1485/3999 |
| Prevalence (%, 95% CI) | 0.61 (0.33-1.03) |  | 0.64 (0.36-1.07) |  |  |  | 0.63 (0.42-0.91) |
| *Haemaphysalis campanulate* |  |  |  |  |  |  |  |
| NPP/NTP/NTT |  |  |  | 0/122/378 |  |  | 0/122/378 |
| Prevalence (%, 95% CI) * |  |  |  | 0.00(-) |  |  | 0.00(-) |
| *Dermacentor silvarum* |  |  |  |  |  |  |  |
| NPP/NTP/NTT | 3/497/1012 | 8/286/286 |  |  |  |  | 11/783/1298 |
| Prevalence (%, 95% CI) | 0.30 (0.08-0.80) | 2.80 (1.32-5.23) |  |  |  |  | 0.85 (0.45-1.46) |
| *Rhipicephalus microplus* |  |  |  |  |  |  |  |
| NPP/NTP/NTT |  |  | 2/283/527 |  | 3/50/140 | 9/143/143 | 14/476/810 |
| Prevalence (%, 95% CI) |  |  | 0.38 (0.68-1.24) |  | 2.19 (0.58-5.85) | 6.29 (3.13-11.21) | 1.74 (1.00-2.83) |
| Total |  |  |  |  |  |  |  |
| NPP/NTP/NTT | 50/1339/4249 | 62/1236/2846 | 15/1252/2544 | 0/122/378 | 3/50/140 | 15/269/269 | 145/4268/10426 |
| Prevalence (%, 95% CI) | 1.20 (0.90-1.56) | 2.37 (1.83-3.02) | 0.59 (0.35-0.95) | 0.00(-) | 2.19 (0.58-5.85) | 5.58 (3.28-8.82) | 1.45 (1.23-1.74) |

*The prevalence (95 % confidence intervals) was calculated by maximum likelihood estimation (MLE) using the program PooledInfRate. NPP, No. of positive pools; NTP, No. of tick pools; NTT, No, of tested ticks.

**Table S5. Positive rate of relapsing fever *Borrelia* in wild small mammals in six eco-climate regions in China**.

| **Wild small mammals** | |  | **No. positive/ No. tested** | | | | | | **No. total positive /**  **No. total tested (%)** |
| --- | --- | --- | --- | --- | --- | --- | --- | --- | --- |
| **Family/Genus** | **Species** |  | **Northeastern China** | **Inner Mongolia-Xinjiang** | **Northern China** | **Central China** | **Southern China** | **Southwestern China** |  |
| *Cricetidae* |  |  | 0/53 | 0/56 | 3/165 |  |  | 0/33 | **3/307(0.98)** |
| *Allocricetulus* | *Allocricetulus eversmanni* |  |  |  | 0/21 |  |  |  | 0/21(0) |
| *Cricetulus* | *Cricetulus barabensis* |  |  | 0/2 |  |  |  |  | 0/2(0) |
|  | *Cricetulus longicaudatus* |  |  |  | 1/28 |  |  |  | **1/28(3.57)** |
|  | *Cricetulus migratorius* |  |  | 0/50 |  |  |  |  | 0/50(0) |
| *Eothenomys* | *Eothenomys cachinus* |  |  |  |  |  |  | 0/1 | 0/1(0) |
|  | *Eothenomys eleusis* |  |  |  |  |  |  | 0/8 | 0/8(0) |
|  | *Eothenomys miletus* |  |  |  |  |  |  | 0/16 | 0/16(0) |
|  | *Eothenomys proditor* |  |  |  |  |  |  | 0/8 | 0/8(0) |
| *Microtus* | *Microtus fortis* |  | 0/5 |  |  |  |  |  | 0/5(0) |
| *Myodes* | *Myodes rufocanus* |  | 0/1 |  | 2/73 |  |  |  | **2/74(2.70)** |
|  | *Myodes rutilus* |  | 0/47 |  |  |  |  |  | 0/47(0) |
| *Phodopus* | *Phodopus roborovskii* |  |  | 0/4 |  |  |  |  | 0/4(0) |
| *Tscherskia* | *Tscherskia triton* |  |  |  | 0/43 |  |  |  | 0/43(0) |
| *Dipodidae* |  |  |  | 0/24 |  |  |  |  | 0/24(0) |
| *Allactaga* | *Allactaga sibirica* |  |  | 0/19 |  |  |  |  | 0/19(0) |
| *Dipus* | *Dipus sagitta* |  |  | 0/5 |  |  |  |  | 0/5(0) |
| *Muridae* |  |  | 0/175 | 25/475 | 12/1319 | 0/16 | 0/68 | 0/325 | **37/2378(1.56)** |
| *Apodemus* | *Apodemus agrarius* |  | 0/16 |  | 1/224 | 0/4 |  |  | **1/244(0.41)** |
|  | *Apodemus chevrieri* |  |  |  |  |  |  | 0/56 | 0/56(0) |
|  | *Apodemus draco* |  |  |  | 1/72 |  |  |  | **1/72(1.39)** |
|  | *Apodemus ilex* |  |  |  |  |  |  | 0/80 | 0/80(0) |
|  | *Apodemus peninsulae* |  | 0/8 |  | 0/73 |  |  |  | 0/81(0) |
| *Bandicota* | *Bandicota indica* |  |  |  |  |  | 0/14 |  | 0/14(0) |
| *Berylmys* | *Berylmys bowersi* |  |  |  |  |  |  | 0/1 | 0/1(0) |
| *Chiropodomys* | *Chiropodomys gliroides* |  |  |  |  |  |  | 0/1 | 0/1(0) |
| *Melomys* | *Melomys burtoni* |  |  |  |  |  |  | 0/2 | 0/2(0) |
| *Meriones* | *Meriones meridianus* |  |  | 0/57 |  |  |  |  | 0/57(0) |
|  | *Meriones unguiculatus* |  |  | 2/181 |  |  |  |  | **2/181(1.10)** |
|  | *Meriones libycus* |  |  | 4/42 |  |  |  |  | **4/42(9.52)** |
| *Micromys* | *Micromys minutus* |  |  |  |  |  |  | 0/1 | 0/1(0) |
| *Mus* | *Mus musculus* |  | 0/9 | 0/97 | 1/348 | 0/3 |  |  | **1/457(0.22)** |
|  | *Mus pahari* |  |  |  |  |  |  | 0/6 | 0/6(0) |
| *Niviventer* | *Niviventer andersoni* |  |  |  | 0/2 |  |  | 0/4 | 0/6(0) |
|  | *Niviventer confucianus* |  |  |  | 1/22 |  |  |  | **1/22(4.55)** |
|  | *Niviventer coxingi* |  |  |  |  |  |  | 0/7 | 0/7(0) |
|  | *Niviventer niviventer* |  |  |  | 7/147 |  |  |  | **7/147(4.76)** |
| *Rattus* | *Rattus andamanensis* |  |  |  |  |  | 0/11 |  | 0/11(0) |
|  | *Rattus brunneusculus* |  |  |  |  |  |  | 0/7 | 0/7(0) |
|  | *Rattus norvegicus* |  | 0/142 | 0/23 | 0/163 | 0/2 | 0/38 |  | 0/368(0) |
|  | *Rattus pyctoris* |  |  | 0/1 |  | 0/1 |  |  | 0/2(0) |
|  | *Rattus steini* |  |  |  |  |  |  | 0/7 | 0/7(0) |
|  | *Rattus tanezumi* |  |  |  | 1/268 | 0/6 | 0/5 | 0/144 | **1/423(0.24)** |
|  | *Rattus yunnensis* |  |  |  |  |  |  | 0/9 | 0/9(0) |
| *Rhombomys* | *Rhombomys opimus* |  |  | 19/74 |  |  |  |  | **19/74(25.68)** |
| *Sciuridae* |  |  | 0/2 | 0/84 | 0/5 |  |  | 0/3 | 0/94(0) |
| *Callosciurus* | *Callosciurus erythraeus* |  |  |  | 0/5 |  |  |  | 0/5(0) |
| *Spermophilus* | *Spermophilus dauricus* |  |  | 0/28 |  |  |  |  | 0/28(0) |
|  | *Spermophilus erythrogenys* |  |  | 0/31 |  |  |  |  | 0/31(0) |
|  | *Spermophilus undulatus* |  |  | 0/25 |  |  |  |  | 0/25(0) |
| *Tamias* | *Tamias sibiricus* |  | 0/2 |  |  |  |  |  | 0/2(0) |
| *Tamiops* | *Tamiops swinhoei* |  |  |  |  |  |  | 0/3 | 0/3(0) |
| *Spalacidae* |  |  |  | 0/47 |  |  |  |  | 0/47(0) |
| *Myospalax* | *Myospalax aspalax* |  |  | 0/2 |  |  |  |  | 0/2(0) |
|  | *Myospalax psilurus* |  |  | 0/45 |  |  |  |  | 0/45(0) |
| **Total** |  |  | **0/230**  **(0)** | **25/686**  **(3.64)** | **15/1489**  **(1.01)** | **0/16**  **(0)** | **0/68**  **(0)** | **0/361**  **(0)** | **40/2850**  **(1.40)** |

**Table S6. The positive number pools of relapsing fever *Borrelia* species by tick species and** **eco-climate regions in China.**

|  | ***Borrelia miyamotoi*** | ***Borrelia theileri*** | ***Borrelia sp.*** |
| --- | --- | --- | --- |
| **Northeastern China** |  |  |  |
| *Dermacentor silvarum* | 2 | 0 | 1 |
| *Haemaphysalis concinna* | 2 | 0 | 0 |
| *Haemaphysalis japonica* | 4 | 0 | 2 |
| *Haemaphysalis longicornis* | 8 | 0 | 4 |
| *Ixodes persulcatus* | 26 | 0 | 1 |
| **Inner Mongolia-Xinjiang** |  |  |  |
| *Dermacentor silvarum* | 7 | 0 | 1 |
| *Haemaphysalis concinna* | 0 | 0 | 48 |
| *Ixodes persulcatus* | 3 | 0 | 3 |
| **Northern China** |  |  |  |
| *Haemaphysalis longicornis* | 11 | 0 | 2 |
| *Rhipicephalus microplus* | 1 | 1 | 0 |
| **Central China** |  |  |  |
| *Haemaphysalis campanulate* | 0 | 0 | 0 |
| **Southern China** |  |  |  |
| *Rhipicephalus microplus* | 0 | 3 | 0 |
| **Southwestern China** |  |  |  |
| *Ixodes ovatus* | 0 | 6 | 0 |
| *Rhipicephalus microplus* | 0 | 9 | 0 |

**Table S7. The positive number of relapsing fever *Borrelia* species in wild small mammals stratified by species of mammals and eco-climate regions in China.**

|  | ***Borrelia miyamotoi*** | ***Borrelia persica*** | ***Borrelia sp.*** |
| --- | --- | --- | --- |
| **Inner Mongolia-Xinjiang** |  |  |  |
| *Allactaga sibirica* | 0 | 0 | 0 |
| *Cricetulus barabensis* | 0 | 0 | 0 |
| *Cricetulus migratorius* | 0 | 0 | 0 |
| *Dipus sagitta* | 0 | 0 | 0 |
| *Meriones libycus* | 0 | 4 | 0 |
| *Meriones meridianus* | 0 | 0 | 0 |
| *Meriones unguiculatus* | 0 | 0 | 2 |
| *Mus musculus* | 0 | 0 | 0 |
| *Myospalax aspalax* | 0 | 0 | 0 |
| *Myospalax psilurus* | 0 | 0 | 0 |
| *Phodopus roborovskii* | 0 | 0 | 0 |
| *Rattus norvegicus* | 0 | 0 | 0 |
| *Rattus pyctoris* | 0 | 0 | 0 |
| *Rhombomys opimus* | 0 | 19 | 0 |
| *Spermophilus dauricus* | 0 | 0 | 0 |
| *Spermophilus erythrogenys* | 0 | 0 | 0 |
| *Spermophilus undulatus* | 0 | 0 | 0 |
| **Northern China** |  |  |  |
| *Allocricetulus eversmanni* | 0 | 0 | 0 |
| *Apodemus agrarius* | 1 | 0 | 0 |
| *Apodemus draco* | 0 | 0 | 1 |
| *Apodemus peninsulae* | 0 | 0 | 0 |
| *Callosciurus erythraeus* | 0 | 0 | 0 |
| *Cricetulus longicaudatus* | 0 | 0 | 1 |
| *Mus musculus* | 0 | 0 | 1 |
| *Myodes rufocanus* | 0 | 0 | 2 |
| *Niviventer andersoni* | 0 | 0 | 0 |
| *Niviventer confucianus* | 0 | 0 | 1 |
| *Niviventer niviventer* | 0 | 0 | 7 |
| *Rattus tanezumi* | 0 | 0 | 1 |
| *Tscherskia triton* | 0 | 0 | 0 |

No existence of RF *Borrelia* species was determined in four eco-climate regions that are not listed in the table: Northeastern China, Central China, Southern China, Southwestern China

**References**

1. Mangold AJ, Bargues MD, Mas-Coma S. Mitochondrial 16S rDNA sequences and phylogenetic relationships of species of Rhipicephalus and other tick genera among Metastriata (Acari: Ixodidae). Parasitology research. 1998 Jun;84(6):478-84.

2. Nicolas V, Querouil S, Verheyen E, Verheyen W, Mboumba JF, Dillen M, et al. Mitochondrial phylogeny of African wood mice, genus Hylomyscus (Rodentia, Muridae): implications for their taxonomy and biogeography. Mol Phylogenet Evol. 2006 Mar;38(3):779-93.

3. Hovius JW, de Wever B, Sohne M, Brouwer MC, Coumou J, Wagemakers A, et al. A case of meningoencephalitis by the relapsing fever spirochaete Borrelia miyamotoi in Europe. Lancet (London, England). 2013 Aug 17;382(9892):658.

4. Jiang BG, Wu AQ, Jiang JF, Yuan TT, Xu Q, Lv CL, et al. Molecular Detection of Novel Borrelia Species, Candidatus Borrelia javanense, in Amblyomma javanense Ticks from Pangolins. Pathogens (Basel, Switzerland). 2021 Jun 9;10(6).

5. Jiang BG, Jia N, Jiang JF, Zheng YC, Chu YL, Jiang RR, et al. Borrelia miyamotoi Infections in Humans and Ticks, Northeastern China. Emerg Infect Dis. 2018 Feb;24(2):236-41.

6. Picken RN. Polymerase chain reaction primers and probes derived from flagellin gene sequences for specific detection of the agents of Lyme disease and North American relapsing fever. Journal of clinical microbiology. 1992 Jan;30(1):99-114.

7. Takano A, Fujita H, Kadosaka T, Konnai S, Tajima T, Watanabe H, et al. Characterization of reptile-associated Borrelia sp. in the vector tick, Amblyomma geoemydae, and its association with Lyme disease and relapsing fever Borrelia spp. Environmental microbiology reports. 2011 Oct;3(5):632-7.

8. Safdie G, Farrah IY, Yahia R, Marva E, Wilamowski A, Sawalha SS, et al. Molecular characterization of Borrelia persica, the agent of tick borne relapsing fever in Israel and the Palestinian Authority. PloS one. 2010 Nov 24;5(11):e14105.
